# Surface-Bioengineered Extracellular Vesicles Seeking Molecular Biotargets in Lung Cancer Cells

**DOI:** 10.1021/acsami.4c04265

**Published:** 2024-06-13

**Authors:** Agata Kowalczyk, Damian Dziubak, Artur Kasprzak, Kamil Sobczak, Monika Ruzycka-Ayoush, Magdalena Bamburowicz-Klimkowska, Sławomir Sęk, Ivan Rios-Mondragon, Teresa Żołek, Elise Runden-Pran, Sergey Shaposhnikov, Mihaela Roxana Cimpan, Maria Dusinska, Ireneusz P. Grudzinski, Anna M. Nowicka

**Affiliations:** † Faculty of Chemistry, University of Warsaw, Pasteura Str. 1, Warsaw PL-02-093, Poland; ‡ Faculty of Chemistry, Biological and Chemical Research Centre, University of Warsaw, Żwirki i Wigury 101 Street, Warsaw PL-02-089, Poland; § Faculty of Chemistry, Warsaw University of Technology, Noakowskiego Str. 3, Warsaw 00-664, Poland; ∥ Department of Toxicology and Food Science, Faculty of Pharmacy, Medical University of Warsaw, Banacha Str. 1, Warsaw PL-02-097, Poland; ⊥ Biomaterials - Department for Clinical Dentistry, University of Bergen, Årstadveien 19, Bergen 5009, Norway; # Department of Organic and Physical Chemistry, Faculty of Pharmacy, Medical University of Warsaw, Banacha Str. 1, Warsaw PL-02-097, Poland; ∇ Health Effects Laboratory, Department of Environmental Chemistry, Norwegian Institute for Air Research, Kjeller 2007, Norway; ○ Norgenotech AS, Gaustadalleen Str. 21, Oslo 0349, Norway

**Keywords:** extracellular vesicle bioengineering, model lipid membrane, lung cancer cells, NUDE mice, preclinical safety

## Abstract

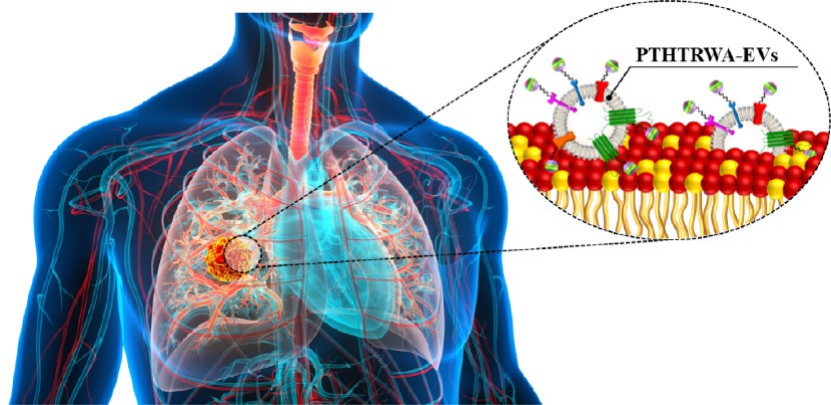

Personalized medicine is a new approach to modern oncology. Here, to facilitate the application of extracellular vesicles (EVs) derived from lung cancer cells as potent advanced therapy medicinal products in lung cancer, the EV membrane was functionalized with a specific ligand for targeting purposes. In this role, the most effective heptapeptide in binding to lung cancer cells (PTHTRWA) was used. The functionalization process of EV surface was performed through the C- or N-terminal end of the heptapeptide. To prove the activity of the EVs functionalized with PTHTRWA, both a model of lipid membrane mimicking normal and cancerous cell membranes as well as human adenocarcinomic alveolar basal epithelial cells (A549) and human normal bronchial epithelial cells (BEAS-2B) have been exposed to these bioconstructs. Magnetic resonance imaging (MRI) showed that the as-bioengineered PTHTRWA-EVs loaded with superparamagnetic iron oxide nanoparticle (SPIO) cargos reach the growing tumor when dosed intravenously in NUDE Balb/c mice bearing A549 cancer. Molecular dynamics (MD) in silico studies elucidated a high affinity of the synthesized peptide to the α5β1 integrin. Preclinical safety assays did not evidence any cytotoxic or genotoxic effects of the PTHTRWA-bioengineered EVs.

## 1 Introduction

Extracellular vesicles are small membrane-bound vesicles secreted by various cell types, including immune cells, stem cells, and cancer cells. These vesicles range in size from 30 to 150 nm in diameter and are composed of lipids, proteins, and nucleic acids.^1^ EVs are formed through the inward budding of the endosomal membrane, resulting in the formation of multivesicular bodies (MVBs) containing intraluminal vesicles (ILVs). These MVBs can then fuse with the plasma membrane, releasing the ILVs into the extracellular environment as exosomes.^2^ Extracellular vesicles play important roles in intracellular communication and are thought to function in a variety of physiological and pathological processes, including immune modulation, tissue regeneration, and cancer progression.^3^ They also can transfer a variety of molecular cargos, including proteins, lipids, and nucleic acids, between the cells altering the phenotype and behavior of recipient cells.^4^ The protein content of extracellular vesicles is highly diverse, and it can vary depending on the cell type that secretes them.^5−8^ Lipids in EVs include phospholipids, cholesterol, and sphingolipids.^9^ The nucleic acids in EVs are primarily composed of RNA species, including mRNA, microRNA, and other small noncoding RNA species.^9^ Extracellular vesicles are capable of crossing biological barriers such as the blood–brain barrier and can deliver their cargo to specific target cells.^4,9,10^ Due to their ability to transfer biologically active molecules, EVs have garnered significant interest in recent years as potential therapeutic agents, targeted transporters, or diagnostic tools, especially based on liquid biopsies.^11,12^ Moreover, extracellular vesicles can be used with other cancer therapies, such as chemotherapy or radiation therapy, so-called combination therapy, enhancing efficacy and reducing side effects compared to the monotherapy approaches.^10,13−17^

To improve the specificity and efficiency of EV-based therapeutics, making them promising tools, e.g., for targeted drug delivery^18^ and personalized medicine^19^ or for imaging to visualize cancer cells and/or solid tumors,^20^ the surface of EVs can be precisely functionalized with specific ligands or antibodies that bind to specific receptors on the target cells. There are many common methods for EV surface functionalization, including covalent and noncovalent conjugation, lipid membrane modification, and genetic engineering.^16,21^ The choice of method for extracellular vesicle surface functionalization depends on their further application and the properties of the ligands or antibodies being used. Successful functionalization can improve the specificity and efficiency of EV-based therapeutics, making them promising tools for targeted drug delivery and personalized medicine.^9^ By modifying the extracellular vesicle surface with targeting moieties, such as antibodies or peptides, the EVs can selectively bind to cancer cells to deliver their cargo and stimulate the immune system, resulting in enhanced efficacy of cancer-killing, reduced toxicity, and improved patient outcomes. Furthermore, in cancer diagnosis, EVs can be isolated from various body fluids, including blood, urine, and cerebrospinal fluid, and their cargo can be analyzed to identify biomarkers for cancer diagnosis and monitoring.^19,22,23^ The multitude of EV functions relates not only to the physiological state but also to disease, so the medical world is open to the benefits that can be achieved through the skillful use of these nanostructures. Particularly high hopes lie in properly functionalized EVs, which have great potential for cancer treatment and diagnosis, and their development is an active area of research in personalized cancer medicine.

Here, we designed and bioengineered extracellular vesicles collected from lung cancer cells. The extracellular vesicle membrane was decorated with a targeting molecule, heptapeptide PTHTRWA, the most effective peptide in binding to lung cancer cells compared with normal lung epithelial cells and different nonlung tumor cells. The functionalization process was based on the 1,3-dipolar cycloaddition reaction, namely, the *click-chemistry* reaction. The as-modified EVs were qualitatively and quantitatively characterized using complementary techniques such as NMR spectroscopy, transmission electron microscopy (TEM), zeta potential (ZP), and dynamic light scattering (DLS). Using attenuated total reflectance infrared spectroscopy (ATR-IR) and surface plasmon resonance (SPR), we specified the activity of the EVs functionalized with PTHTRWA in binding to the normal and cancer model lipid membrane as well as human lung cancer cells compared with human normal bronchial epithelial cells. These effects were also confirmed by MRI studies when the PTHTRWA-EVs loaded with SPIO cargos were intravenously dosed in NUDE Balb/c mice bearing human A549 lung cancer. Preclinical safety assays did not elucidate any cytotoxic and genototoxic effects of these novel bioconstructs, although the impedance-based studies showed some transient effects on the endothelial barrier integrity. Computational studies provide evidence that the α5β1 integrin is a molecular target for the PTHTRWA-EVs in lung cancer.

## 2 Experimental Setup

### 2.1 Materials

*N*-(*tert*-butoxycarbonyl)-l-alanine (Boc-Ala-OH), *N*_α_-(*tert*-butoxycarbonyl)-l-tryptophan (Boc-Trp-OH), *N*_α_-(*tert*-butoxycarbonyl)-l-arginine (Boc-Arg-OH), *N*-(*tert*-butoxycarbonyl)-l-threonine (Boc-Thr-OH), *N*_α_-(*tert*-butoxycarbonyl)-l-histidine (Boc-His-OH), *N*-(*tert*-butoxycarbonyl)-l-proline (Boc-Pro-OH), cesium hydrogen carbonate, trifluoroacetic acid (TFA), *N*,*N*′-dicyclohexylcarbodiimide (DCC), Merrifield resin (4.1 mmol·g^–1^ Cl^–^ loading; 100–200 mesh), triethylamine (TEA), methyl-*tert*-butyl ether (MTBE), (2*R*,3*R*)-1,4-dimercapto-2,3-butanediol (DTT), hydrobromic acid (HBr; 48%), glacial acetic acid (AcOH), ethanol (99.8%, EtOH), *N*,*N*-dimethyformamide (DMF), trifluoroacetic acid (TFA), 1-ethyl-3-(3-carbodiimide dimethylaminopropyl)carbodiimide (EDC), *N*-hydroxysuccinimide (NHS), 4-pentynoic acid (4-PA), 6-azido-hexanoic acid (6-AHA), 3-azido-1-propanamine (3-APA), copper(II) sulfate pentahydrate (CuSO_4_·5H_2_O), ascorbic acid (AA), bathophenanthrolinedisulfonic acid disodium salt hydrate (BPDSA), 1,2-dimyristoyl-*sn*-glycero-3-phosphocholine (DMPC), cholesterol (Chol), 1,2-dimyristoyl-*sn*-glycero-3-phospho-l-serine (DMPS), hydrofluoric acid (HF), Dulbecco’s modified Eagle medium (DMEM, Gibco) supplemented with GlutaMAX (Gibco), fetal bovine serum (FBS, Gibco), MCDB131 (Gibco) medium supplemented with GlutaMAX and FBS, penicillin, streptomycin, LHC-9 medium (Gibco), human fibronectin, bovine serum albumin, and hydrocortisone were purchased from Sigma-Aldrich and used without additional purification. Human collagen type I was purchased from Advanced Biomatrix, USA, and human epidermal growth factor was from Miltenyi Biotec, Germany.

### 2.2 Cell Culturing

The cell lines A549 (adenocarcinoma human alveolar basal epithelial cells), BEAS-2B (normal human bronchial epithelial cells), and HULEC-5a (human microvascular endothelial cells) were purchased from ATCC (USA). A549 cells were cultured in DMEM (Gibco) supplemented with GlutaMAX (Gibco), 10% FBS (Gibco) and 10 U·mL^–1^ penicillin–100 μg·mL^–1^ streptomycin. BEAS-2B was cultured in LHC-9 medium (Gibco) on culture flasks precoated with a mixture of 0.01 mg·mL^–1^ human fibronectin, 0.03 mg·mL^–1^ human collagen type I, and 0.01 mg·mL^–1^ bovine serum albumin. HULEC-5a was cultured in MCDB131 (Gibco) medium supplemented with GlutaMAX, 10% FBS, 10 ng·mL^–1^ human epidermal growth factor, and 1 μg·mL^–1^ hydrocortisone. All cells were maintained at 37 °C and 5% CO_2_ in vented cell culture flasks and subcultured when reaching ∼80% confluency. Cells were used at passage numbers below 13 and viability >95%.

### 2.3 Heptapeptide H_2_N-Pro-Thr-His-Thr-Arg-Trp-Ala-OH Synthesis

The heptapeptide OH-Ala-Trp-Arg-Thr-His-Thr-Pro-NH_2_ (PTHTRWA) was synthesized in 9 steps, employing the solid phase peptide synthesis (SSPS) methodology.^24−26^ The detailed designed protocol is described in the Supporting Information, Section 1. The composition of the peptide was confirmed on acidic hydrolysis by analyzing amino acid silyl derivatives using GC–MS analysis (see Table S1 and Figure S1 in the Supporting Information, Section 2).

### 2.4 Bioconjugation of Extracellular Vesicles with Targeting PTHTRWA

**Figure 1:**
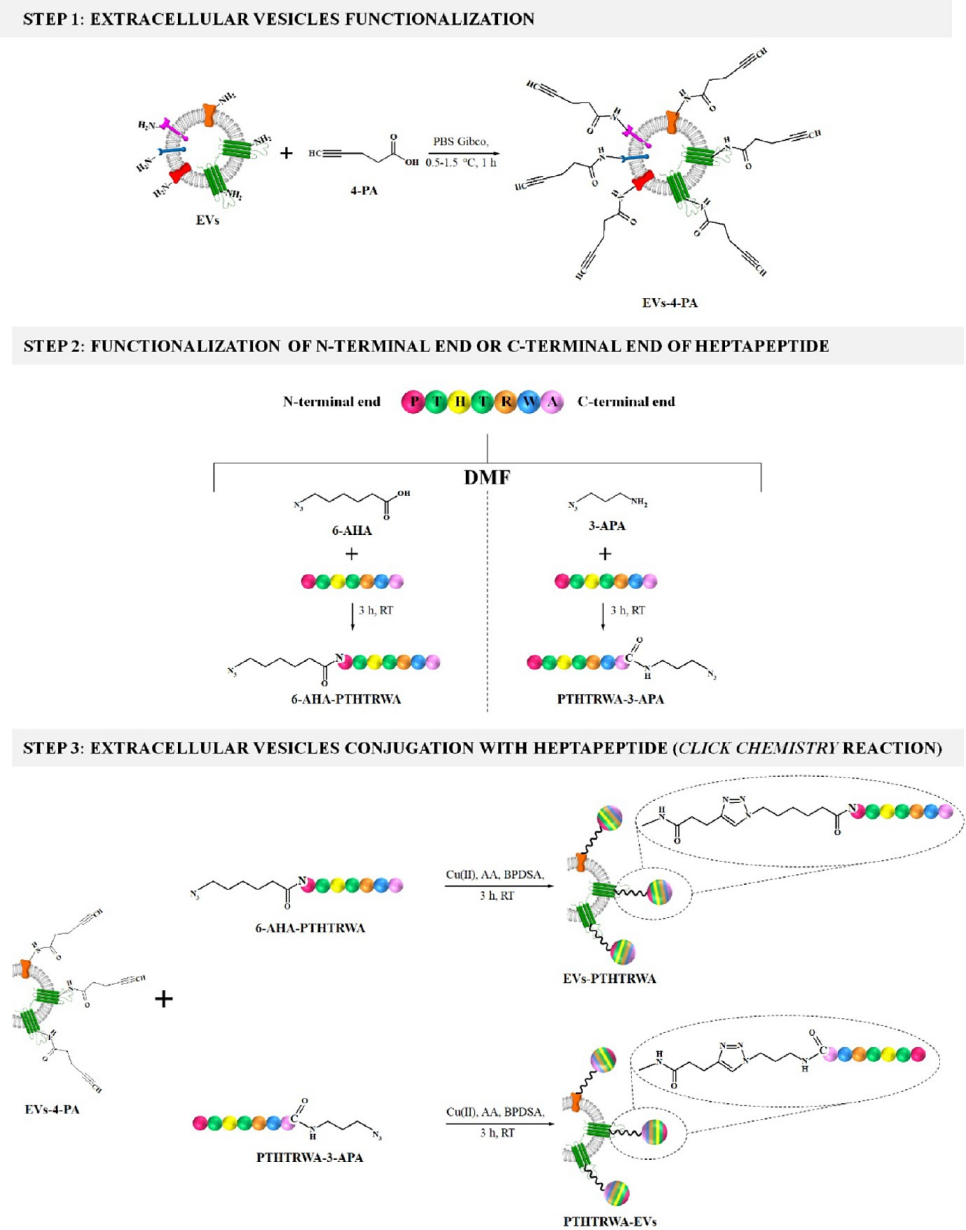
Scheme of the bioconjugation process of EVs with heptapeptide.

The covalent decoration of the extracellular vesicles surface with targeting ligand, namely, the peptide sequence PTHTRWA specific for the A549 cell line, was performed using the 1,3-dipolar cycloaddition reaction, that is, *click chemistry* reaction. The wide employment of *click chemistry* processes in designing the bioprobes results from conducting such reactions fast at mild conditions, as well as from their high selectivity and shows compatibility in aqueous media.^27−30^ The scheme of the bioconjugation process is presented in Figure 1.

*Step* 1: the carboxylic groups of 4-pentynoic acid molecules were activated by adding 0.3 mmol of 4-PA in 1 mL of PBS Gibco buffer pH 7.4 (pH was adjusted to the value of 7.4 with sodium bicarbonate) and an equimolar amount of NHS. The obtained mixture was stirred at a temperature of 0.5–1.5 °C (ice bath) for 1 h. Next, 0.3 mmol of EDC was added to the reaction mixture and stirred in the ice bath at 0.5–1.5 °C for 1 h. Then, 27 μL of the obtained mixture (activated 4-PA) was added to 160 μg of extracellular vesicles (weight based on protein content) in 1 mL of PBS buffer and stirred for 24 h at room temperature. The extracellular vesicles modified with 4-PA (EVs-4-PA) were purified from excess reaction reagents by triple dialysis performed in PBS Gibco buffer using Spectra/Por Dialysis Membrane MWCO: 100–500 Da. The EV isolation and identification steps were described in the Supporting Information, Section 3.

*Step* 2: the heptapeptide can be conjugated with extracellular vesicles through an N-terminal or C-terminal amino acid. So, the second step of the bioconjugation reaction concerned the functionalization of the N-terminal end of the peptide with the 6-azido-hexanoic acid or C-terminal end of the peptide with 3-azido-1-propanamine. In the case of the functionalization of the N-terminal amino acid of the peptide, the first step was the activation of the carboxylic groups of 6-AHA using solid DCC. To 1 mL of 6-AHA (0.0127 mmol in DMF), 0.0138 mmol of solid DCC was added and left to react for 30 min at room temperature. Next, 1 mL of heptapeptide (10 mg·mL^–1^ ≡ 0.0115 M) in DMSO was added to the reaction mixture and left under stirring at room temperature for 3 h. The functionalization of the C-terminal amino acid of the heptapeptide with 3-APA included a single step involving the mixing of 1 mL of the heptapeptide (10 mg·mL^–1^ ≡ 0.0115 M), 0.012 mmol 3-APA, and 0.0138 mmol DCC and left under stirring at room temperature for 3 h.

*Step* 3: the conjugation of EVs-4-PA with the 6-AHA-PTHTRWA or PTHTRWA-3-APA peptide was performed using the *click chemistry* approach. For this purpose the following reagents: 160 μg of EVs-4-PA (weight based on protein content), 0.23 μmol of N-terminated heptapeptide (6-AHA-PTHTRWA) or C-terminated heptapeptide (PTHTRWA-3-APA), 15 μmol of CuSO_4_·5H_2_O, 340 μmol of ascorbic acid (AA), 29.5 μmol of bathophenanthrolinedisulfonic acid disodium salt hydrate were mixed and left under the stirring at room temperature for 4 h. The obtained solutions, EVs-PTHTRWA and PTHTRWA-EVs were dialyzed (at least 3 times) against Gibco buffer pH 7.2 to remove unbound reagents.

Quantitative characterization of EV surface bioengineering with heptapeptide was based on protein content quantified using a BCA protein assay. In calculations, an average exosomal protein molecular weight of 150 kDa (149947 g·mol^–1^) was assumed. Nonfunctionalized EVs in concentration 3.82 × 10^9^ particles·mL^–1^ contain 160 μg of protein, which is equivalent to 1.07 × 10^–9^ mol and 6.43 × 10^14^ molecules. After EV functionalization with heptapeptide through the C-terminus end, the protein content increased to the value 540 μg·mL^–1^. Assuming an analogous calculations and a PTHTRWA-EV concentration of 1.92 × 10^9^ particles·mL^–1^, the number of heptapeptide molecules per EV protein is 7. In turn, in the case of EVs-PTHTRWA (functionalization through the N-terminus end) at a concentration of 0.52 × 10^9^ particles·mL^–1^ (containing 270 μg of protein), the number of heptapeptide molecules per EV protein is 3.5.

### 2.5 Applied Characterization Methods

Attenuated total reflectance infrared spectroscopy experiments were performed with a Nicolet iS50 FTIR spectrometer (Thermo Fisher Scientific, Waltham, MA, USA) with an MCT-A detector (cooled with liquid nitrogen) and a custom-made single-reflection accessory. A hemisphere silicon prism was used as a substrate for further examination of the interaction between the EVs and the model lipid bilayer. The substrate was cleaned before use. First, the silicon prism was polished on the 3 polishing cloths with suspensions containing 3 different sizes of diamond particles (3.0, 1.0, and 0.25 μm). Then, the prism was washed with pure Mili-Q water, placed in a crystallizer with ethanol, and sonicated for 30 min. Directly before use, on the top of the hemisphere silicon prism, 1 mL of 2% HF was dropped and left for 3 min. After that, the prism was washed again with pure Milli-Q water and placed in the UV chamber for 30 min. Such a prepared and cleaned prism was directly mounted onto the homemade accessory for further experiments. The creation of the biomimetic lipid membrane was performed using rapid solvent exchange. The mixture of DMPC and Chol in a molar ratio of 7:3 was used as a lipid composition, representing the eukaryotic “health” cell membrane, while DMPC and DMPS in a molar ratio of 7:3 were supposed to mimic the lipid composition of the cancer cell membrane. All lipids were dissolved in chloroform. The final concentration of the lipid mixtures was 1 mM. The first step during the experiments was collecting the background spectra. In such a case, 1 mL of buffer was added to the ATR-IR cell with an already mounted clean silicon hemispherical prism. After 30 min, the background (*I*_0_) spectra were collected, and the buffer was removed. Then, 1 mL of the lipid solution was added and left for 5 min, while not allowing for complete evaporation of the chloroform. Then, the prism was gently washed with buffer until all chloroform was removed. This procedure (repeated washing of the cell with buffer) led to the formation of a membrane. After that, the sample (*I*) spectra of the membrane were collected. In the case of examination of the interaction between the EVs and model lipid membrane, to the already prepared membrane was added 1 mL of a suspension of EVs and left for 2 h. The presented data are displayed as the differential absorbance, calculated according to the formula *A* = log(*I*_0_/*I*), where *I*_0_ is a background (buffer), while *I* is a spectrum recorded in the presence of the membrane either before or after interaction with EVs.

The surface plasmon resonance experiments were performed by using gold sensor chips and a Biacore X100 system (GE Healthcare) from Cytiva (Uppsala, Sweden). Before the measurements, the gold sensor chip was cleaned in the TL1 mixture: ultrapure water, 25% ammonia, and 30% hydrogen peroxide (v:v ratio 5:1:1) at 75 °C for 5 min. Next, the surface of the sensor was rinsed with ultrapure water, followed by ethanol (99.8%), and dried with Ar stream. Measurements were carried out in the flow system using 0.01 M Gibco buffer (pH 7.4) as a running buffer. The surface of the gold sensor chip was modified with the (i) model lipid cell membranes: normal (DMPC: Chol; 7:3) and cancer (DMPC: DMPS; 7:3) and (ii) noncancerous human normal bronchial epithelial cells and cancerous human adenocarcinomic alveolar basal epithelial cells outside the Biacore X100 system.

TEM investigations were carried out by the FEI Talos F200X microscope operated at 200 kV. Morphological and energy-dispersive X-ray spectroscopy (EDX) observations were performed in scanning transmission electron microscopy (STEM) mode using a high-angle annular dark field (HAADF) detector. EDX spectroscopy using a Super-X system with four SDDs was applied to the detection of differences in the local chemical composition. The specimens were stained with a contrast AGR1000 - UA-Zero EM Stain (Agar).^31^

The NMR experiments were carried out using a JEOL 600 MHz spectrometer equipped with a multinuclear z-gradient inverse probe head. The spectra were recorded at 25 °C, and standard 5 mm NMR tubes were used. The spectra were references to the solvent signal, i.e., D_2_O: δ_H_ (residual HOD) 4.79 ppm. The sample preparation was as follows. The dispersion of the nonfunctionalized EVs or EVs decorated with heptapeptide was lyophilized for 48 h. The as-lyophilized material was then dissolved in D_2_O, shaken (700 rpm) for 30 min, and the sample was filtered off using a syringe filter (0.22 μm) directly into the NMR tube.

The dynamic light scattering and zeta potential measurements were carried out in PBS buffer at 25 °C using a Zetasizer nano series apparatus (Malvern Panalytical Ltd., UK) with a He–Ne (4 mW) laser at 632.8 nm. The data were collected during 3 cycles (5 repetitions every 10 s in each cycle).

The preclinical experiments were performed in compliance with the second Local Animal Research Committee, Warsaw University of Life Sciences, Poland (approval No. WAW2/077/2022, dated 22.06.2022) and conducted according to the law for the welfare of animals and regulations for the care and use of laboratory animals. Five-week old male NUDE Balb/c mice purchased from Charles River Laboratories (Germany) were housed in a group of five in individually ventilated cages (IVC) with free access to a standard diet (Altromin) and water ad libitum, and they were placed in 12 h light/dark cycle. The animals were acclimated to the animal facility for at least 1 week prior to the experimental procedure and were injected into the right flank with 4 × 10^6^ A549 cells suspended in 100 μL of the culture medium. On three-week postimplantation, the naïve mice bearing A549 tumors were intravenously injected (0.2 mL) into the tail vail with the as-obtained SPIO-loaded PTHTRWA-EVs (1.0 × 10^11^ EV particles·mL^–1^; ca. 250 mg·mL^–1^) in normal saline and subjected to magnetic resonance imaging to visualize the tumor. MRI was performed using an actively shielded Bruker 7.0 T BioSpin MRI scanner (Bruker Corp, Ettlingen, Germany), with B-GA20S gradient drivers, and a 30 cm USR magnet. T2-weighted morphological images were performed in the Turbo RARE sequence with the following parameters: TR 4500 ms, TE 30 ms, FA 180.0 deg, TA 19 m12 s, FOV 3.20 cm, MTX 256.

### 2.6 Molecular Dynamics In Silico Studies

Molecular dynamics (MD) simulations of the PTHTRWA-receptor complex were performed to investigate the binding of PTHTRWA heptapeptide with the α5β1 integrin and the influence of its structure on the intermolecular interactions. All MD simulations were run using the CHARMm force field^32^ implemented in Discovery Studio v21 interface BIOVIA.^33^ The starting structure of the system was immersed in a rectangular TIP3P water box, in which the receptor atom was at least 20 Å away from the nearest edge of the box.^34^ All systems were immersed in NaCl aqueous salt solution at a concentration of ∼0.15 M, close to physiological conditions (Na^+^ and Cl^–^ ions were randomly added to the water box). Energy minimizations and MD simulations were performed using the particle mesh Ewald (PME) method^35^ for the correct treatment of electrostatic interactions.^36^ To estimate the stability and binding affinity of PTHTRWA to the integrin, we calculated the free energies of the integrin, ligand, and integrin-ligand complex. For this purpose, we used the molecular mechanics/Poisson–Boltzmann surface area (MM/PBSA) method, which followed MD simulations.^37−39^ The detailed computational studies are described in the Supporting Information, Section 4.

### 2.7 Preclinical Safety In Vitro Studies

Electric cell–substrate impedance sensing (ECSIS) was used to assess the impact of the EVs at different concentrations on proliferation and viability of A549 and BEAS-2B cells and endothelial barrier integrity of a monolayer of HULEC-5a cells. This technique monitors the viability, cell number, cell–substrate, and cell–cell contact in real-time. All of these variables affect current flow across the electrode array onto which cells grow. These changes in current flow are measured to determine electrical impedance, which is reported by the system with a dimensionless cell index (CI) value. The CI is directly proportional to the cell surface coverage and integrity of the cell barrier. More details information on this assay and results are provided in the Supporting Information, Section 5.

Cytotoxicity and genotoxicity of the EVs were investigated in the two cell lines including A549 cells and BEAS-2B cells by the Alamar Blue. DNA damages were investigated by a single cell gel electrophoresis/comet assay (CA). DNA strand breaks (SB) were detected by the standard alkaline comet assay, while oxidized base lesions were detected with the modified version of the assay using the enzyme formamidopyrimidine (Fpg) DNA glycosylase (kind gift from NorGenoTec AS, Norway). More detailed information on these assays and results is provided in the Supporting Information, Section 5.

### 2.8 Uptake of DiOc18(3)-Labeled EVs

EVs and PTHTRWA-EVs (1 × 10^8^ EVs) were incubated with 10 μM DiOC18(3) (ThermoFischer Scientific) in PBS containing 0.05% BSA for 30 min at 37 °C under constant and gentle mixing. Staining was stopped by adding BSA at a final concentration of 0.5%. Free and BSA-bound dye were removed by washing three times with PBS (15 mL per wash) using 100 kDa ultra centrifugal filters (Merck Millipore). The EV concentrate was then centrifuged at 20000*g* for 10 min to remove DiOC18(3) micro/nanoparticles. An EV-free PBS solution underwent the same staining procedure to take into account residual free dye (background staining control).

For EV uptake experiments, A549 and BEAS-2B cells were plated in imaging chambers (IBIDI GmbH) at 25000 and 50000 cells·cm^–2^, respectively. Cells were cultured for 24 h at 37 °C and then exposed to DiOC18(3)-labeled EVs or PTHTRWA-EVs for 2 h. Cells were washed twice with warm medium and fixed with 4% paraformaldehyde/0.25% glutaraldehyde in PBS containing 4% sucrose. Cells were washed twice with PBS, and the cell membrane was stained with 50 μg·mL^–1^ wheat germ agglutinin-Alexa Fluor 647 (ThermoFischer Scientific). Specimens were imaged immediately after staining using a TCS SP8 confocal microscope (Leica Microsystems) equipped with a 100× objective (NA = 1.4), resonant scanner, white laser, and hybrid detectors. All specimens were imaged using the same conditions (i.e., laser power, intensity, gain, pinhole size, voxel size, and line average). The open-source software Fiji was used to process the images.^40^

## 3 Results and Discussion

Modern cancer therapy is one of the fastest growing fields of medicine. It is because existing therapeutics, drug delivery systems, or targeted therapies despite continuous improvements are still not perfect. Their greatest, and at the same time most difficult to overcome disadvantage, is the low selectivity of their action only toward the cancer cells and, in consequence, their high toxicity. Therefore, there is a constant need to improve drug delivery systems toward increasing their selectivity against cancer cells. The best way to increase the selectivity of drug delivery systems seems to be the functionalization of the drug carrier with self-navigating molecules, which increases the probability of delivering the drug to the desired place, which allows protection of healthy cells. The tumor cell surface has numerous cancer markers such as tumor-associated antigens (TAAs) or tumor-specific antigens (TSAs), which facilitate the identification of the target site of action of the drug. In our studies on the role of drug delivery system, extracellular vesicles derived from lung cancer cells were used. To improve its selectivity against lung cancer, their surface was functionalized with targeting ligand the heptapeptide sequence PTHTRWA. According to the literature, this peptide sequence increased effectiveness of carrier/drug binding with lung cancer cells compared to normal lung epithelial cells.^41^

### 3.1 Physicochemical Characteristic of EVs Decorated with Heptapeptide

**Figure 2:**
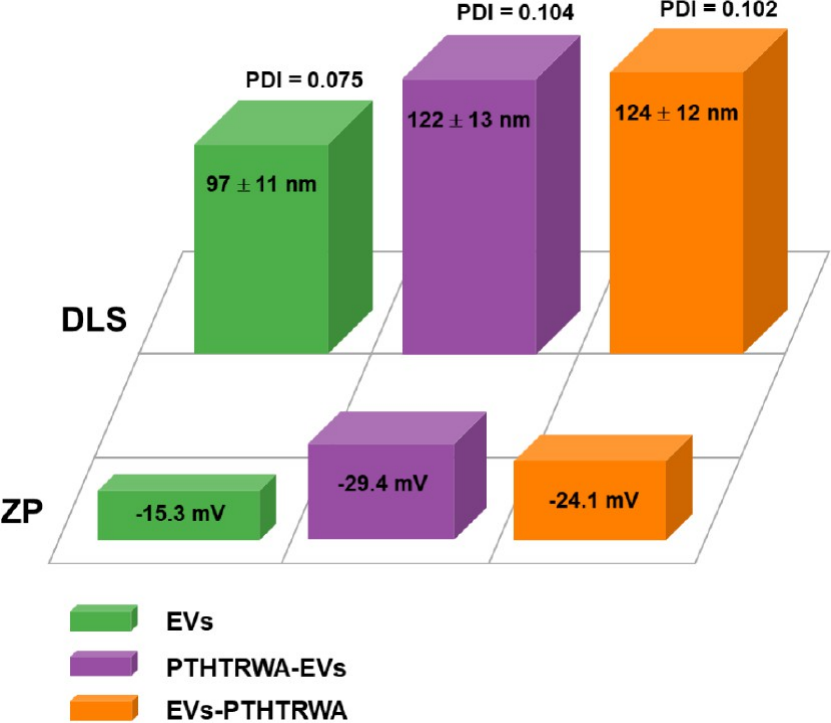
Mean size, PDI, and ZP of nonfunctionalized EVs or EVs decorated with heptapeptide dispersed in PBS buffer based on DLS studies (*n* = 5).

The DLS measurements, presented in Figure 2, demonstrate that the hydrodynamic diameter of EVs increased after the covalent decoration of the EV surface with heptapeptide regardless of whether conjugation took place through C- or N-terminal amino acid. Moreover, the small values of polydispersity index (PDI) (0.075–0.104) testify that the size distribution of nonfunctionalized EVs or EVs decorated with heptapeptide is narrow, which proves that the obtained conjugate solutions are monodisperse and stable (ZP > −20 mV).

**Figure 3:**
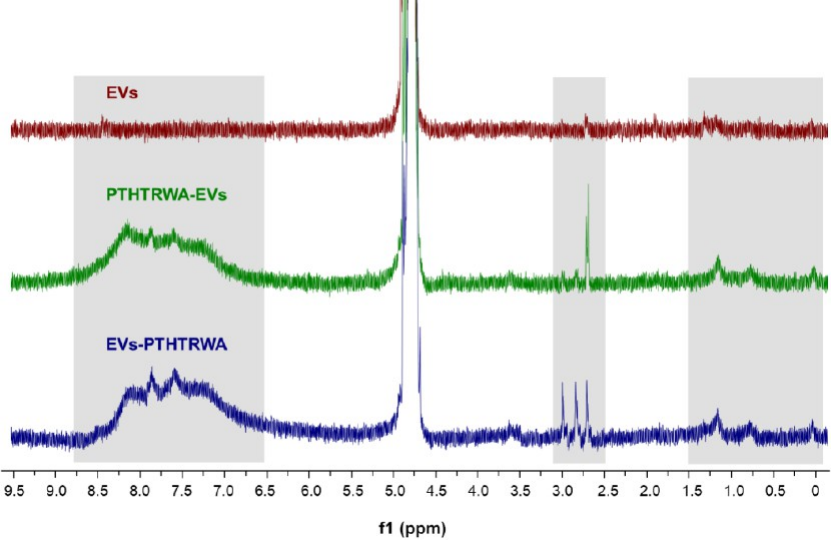
^1^H NMR spectra (600 MHz, D_2_O) of nonfunctionalized EVs (red), PTHTRWA-EVs (green), and EVs-PTHTRWA (blue). The regions of the spectra in which significant differences were observed were marked with gray frames.

The stacked ^1^H NMR spectra of nonfunctionalized EVs or EVs decorated with heptapeptide through N-terminal or C-terminal amino acids (EVs-PTHTRWA and PTHTRWA-EVs) are presented in Figure 3. Despite the signals found in the ^1^H NMR spectra being of low intensity due to the characteristics of such biosample, this experiment supported the surface modification of the EVs. The spectra of EVs-PTHTRWA and PTHTRWA-EVs featured the signals located both in the aromatic (8.50–7.00 ppm) and aliphatic (3.00–2.50 ppm and 1.30–0.10 ppm) region. On the contrary, the ^1^H NMR spectrum of nonfunctionalized EVs featured no significant detectable peaks in these areas. The signals in the aromatic and aliphatic regions in the spectra of functionalized EVs could be ascribed to the protons coming from the amino acids and introduced linkers. *Click chemistry* derived 1,2,3-triazole linkage resulted in the presence of the signals coming from the aromatic C–**H**. The presence of other structural moieties from the linker, i.e., methylene moieties (CH_2_), resulted in the presence of the signals coming from aliphatic C–**H**. In the case of the amino acids in the peptide structure, the presence of Thr and His amino acids resulted in the presence of the signals in the aromatic region, while the signals in the aliphatic regions could be ascribed to the presence of all the amino acids (Ala, Trp, Arg, Thr, His, Pro) in the sample.

**Figure 4:**
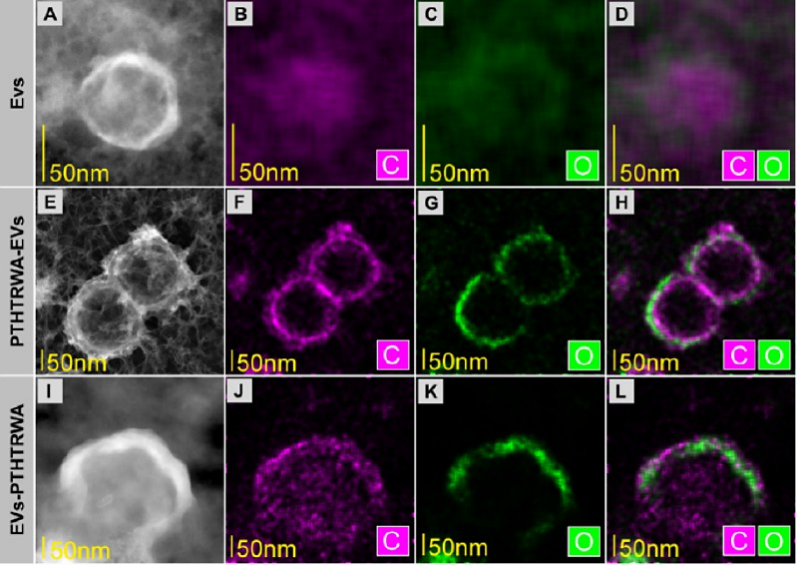
TEM images of nonfunctionalized EVs (A–D), PTHTRWA-EVs (E–H), and EVs-PTHTRWA (I–L).

The exemplary TEM results of both the nonfunctionalized EVs (reference system) and the EVs decorated with heptapeptide from the C end (PTHTRWA-EVs) are presented in Figure 4. Images A and E show EVs made with the HAADF detector (sensitive to atomic number (so-called Z-contrast)).^42^ There is a difference in the appearance of the extracellular vesicles surface, indicating that successful deposition of PTHTRWA on the surface EDX investigation confirmed the existence of a shell containing O and C on the surface of the EVs, which is shown in Figure 4F–H. An attempt to deposit the heptapeptide via the N-end (EVs-PTHTRWA) on the surface of the EVs fared less well (Figure 4I–L). Part of the EVs was damaged.

**Figure 5:**
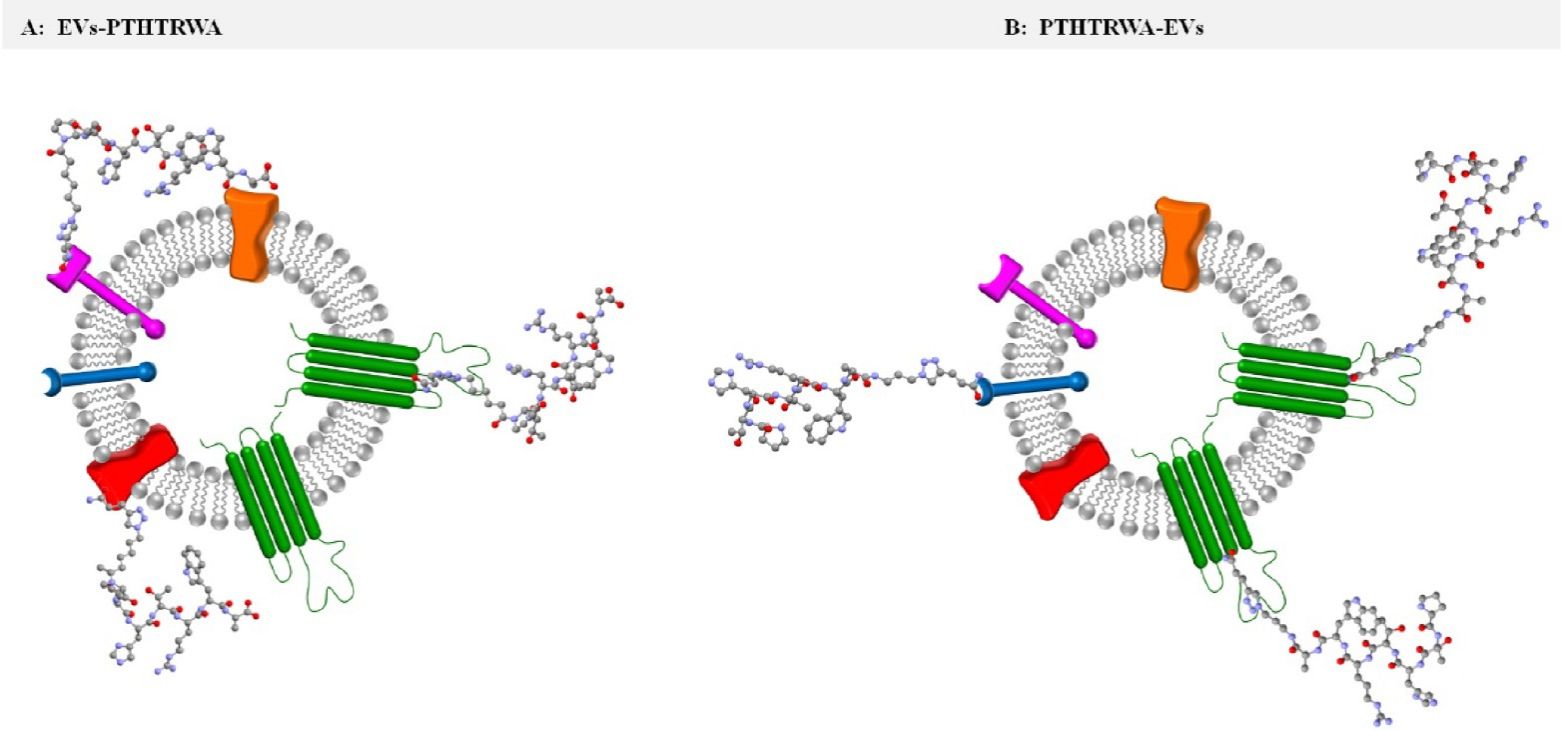
Possible optimized structures of heptapeptide anchored to the EV surface through its N- and C-terminus ends.

Given that the conditions of synthesis as well as chemicals used during the step of extracellular vesicle surface-bioengineering with heptapeptide through its C- or N-terminal end were the same, a likely reason for the partial destruction of some of EVs-PTHTRWA is the orientation of the heptapeptide on the exosome surface. The geometry of the heptapeptide structure with an appropriate linker was optimized using Avogadro software v 1.2.0 (Force field: MMFF94s, number of steps: 500, algorithm: steepest descent). Taking into account the obtained structures, it is evident that structure of heptapeptide linked with EVs through its N-terminal group is more bent, and the shape may resemble a “hook or hairpin”, see Figure 5A. The anchoring to the exosome surface of the most bent (hook-like) structure may contribute to the disruption of the exosome membrane. In contrast, the analysis of the possible structures of the heptapeptide attached through its C-terminus to the EV surface (PTHTRWA-EVs) in each case is more linear, with no apparent bends; see Figure 5B.

### 3.2 Interactions of Extracellular Vesicles Decorated with Targeting Heptapeptide and Normal/Cancer Model Lipid Membranes

Cell membranes are complex assemblies of lipids and proteins that separate the inside of a cell from the outside environment. Thus, they are the first target of any drug.^43^ Drugs can act on the cell membrane surface or have intracellular targets. The intracellular activity of a drug involves its penetration through the membrane into the cell interior. Therefore, understanding at the molecular level, the mechanism of drug interaction with cell membranes is crucial in the design of new therapeutic and drug delivery systems. Biomimetics of cell membranes, lipid bilayers, are widely used models in studies aimed at characterizing the interactions of various types of molecules with the cell membrane, as they perfectly mimic the action of natural biological systems in terms of their functionality and mechanisms of action.^44,45^ In the studies, two models of biological cell membranes (normal and cancer cells) were used. The normal cell membrane consisted of DMPC and Chol (7:3), whereas the cancer cell membrane consisted of DMPC and DMPS (7:3).^46,47^ The experiments were performed by using ATR-IR and SPR techniques.

**Figure 6:**
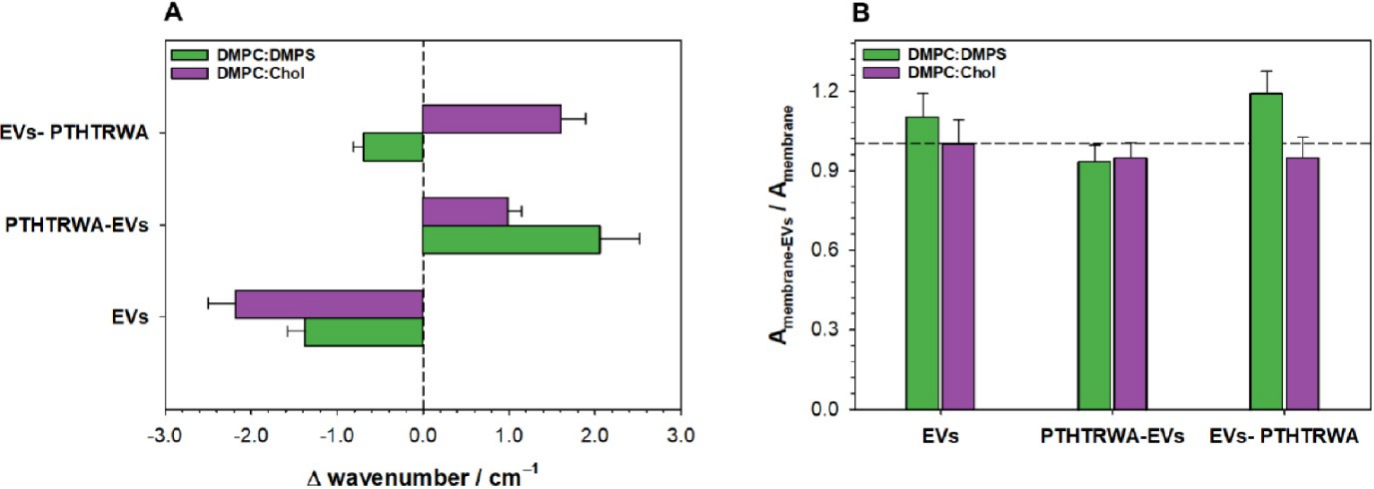
(A) The difference in the position of the υ(CH)_as_ band is between a model membrane of a healthy (DMPC:Chol) and cancer cell (DMPC:DMPS) after interaction with appropriate EVs and the membrane. (B) The ratio of the υ(CH)_as_ band absorbance for the membrane after the interaction with nonfunctionalized and functionalized EVs (EVs, PTHTRWA-EVs, and EVs-PTHTRWA) and the model system of cell membranes of a healthy or cancer cell.

Using IR to monitor cells seems difficult since the complexity of their structure does not allow recording of the IR spectrum with sufficient resolution. However, due to the extremely shallow depth of penetration, the ATR-IR technique offers opportunities to study biological events occurring near or on the cell membrane once it makes contact with the surface of the prism. Figure 6 presents the changes in the υ(CH)_as_ band position and intensity after the interaction of EVs with the model cell membrane. This band reflects changes within the hydrophobic part of the membrane. In general, the increase in absorption intensity might suggest that the acyl chains are more tilted with respect to the surface normal while the decreased absorbance corresponds to the situation when the acyl chains are more parallel to the surface of normal. This is due to the fact that the transition dipole moment of υ(CH)_as_ vibration is perpendicular to the molecular axis of lipid acyl chains, and the absorbance depends on the angle between the vectors of the transition dipole moment of the given vibration and the electric field of the incident beam.^48^ Moreover, the position of the υ(CH)_as_ band (Figure 6A) is directly related to the fluidity of the membrane. The lipid membrane shows greater fluidity when the band is shifted toward higher wavenumbers, while the shift toward lower values indicates that the membrane is more rigid.^46,49,50^ In the presence of nonfunctionalized EVs, both healthy and cancer cell membranes become stiffer, as evident from the υ(CH)_as_, the band shifts toward lower wavenumbers. However, for nonfunctionalized EVs interacting with the normal model membrane, the intensity of these bands (Figure 6B) remains unchanged, whereas in the case of the cancer cell model membrane, an increase in intensity is observed. This suggests that EVs have a consistent impact on membrane fluidity in both cases, while the molecular mechanism behind membrane stiffening differs. In the case of the DMPC/Chol membrane, it is likely that the two effects compensate for each other. Membrane stiffening may indicate increased molecular packing density due to lipid transfer from EVs to the membrane, leading to a higher band intensity. Simultaneously, there may be a decrease in the acyl chain tilt relative to the surface normal, causing a reduction in band intensity. If these two effects offset one another, the absorbance remains unchanged. On the other hand, in the case of the DMPC/DMPS system, there is an increase in absorption intensity, which, in conjunction with membrane stiffening, may indicate the transfer of lipid molecules from vesicles to the membrane. Despite the differences in the lipid composition of the model membranes, the influence of PTHTRWA-EVs is the same. We noticed a positive shift of the band indicative of slightly increased fluidity of the membrane. This is accompanied by a decrease in absorption intensity, which can be explained either by the reorientation of the acyl chain to more parallel to the surface normal or by the partial loss of lipidic material from the membrane. The first scenario would be contradictory to the observed fluidization of the membrane since chain straightening is expected to increase packing density and ordering. Thus, the second interpretation seems to be more reasonable. Such behavior may be explained by PTHTRWA-EVs adsorption on the membrane and the transfer of some fraction of the lipids from the membrane to the vesicles. This way, the packing density of membrane lipids is decreased, leading to increased fluidity of the system. In the case of EVs-PTHTRWA, different behavior of the υ(CH)_as_ band is observed depending on the type of model membrane. The fluidity of the DMPC:DMPS lipid membrane decreases very slightly (small negative shift of the band), while for the DMPC:Chol membrane, it becomes more fluid (positive shift of the band). At the same time, the absorbance for the model of cancer cell membrane is significantly increased, while for the healthy one, the value of absorbance drops. Based on this information, it can be inferred that in the case of the DMPC/DMPS system, the packing density of molecules increases as a result of significant lipid transfer from vesicles to the membrane. This process may be considered as the interdigitation of lipid chains from EVs and the membrane. It is worth noting that in this case the increase in band intensity is the highest observed among all systems, which may indicate the fusion-like character of the interaction. On the other hand, in the case of the DMPC/Chol membrane, the fluidization effect is accompanied by a decrease in absorbance, which can be explained similarly as in the PTHTRWA-EVs system, i.e., we are dealing with adsorption on the membrane and the transfer of a certain fraction of lipids from the membrane to vesicles.

**Figure 7:**
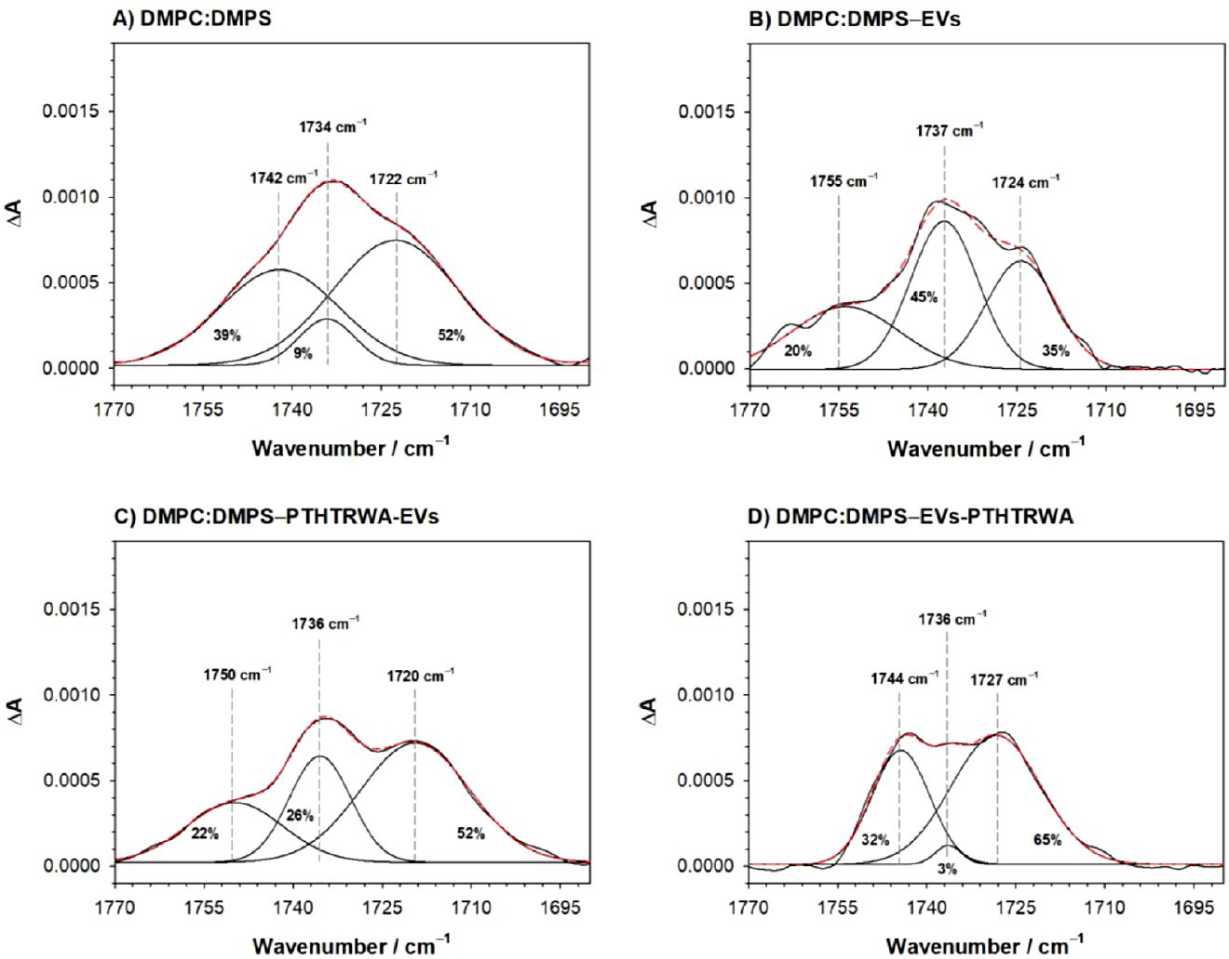
Deconvoluted spectra of the C=O bond for the membrane DMPC:DMPS, what represents the cancer cell membrane (A), and membrane after interaction with nonfunctionalized (B) and appropriately functionalized EVs (C and D).

Further conclusions on the nature of interactions between vesicles and the membranes can be drawn based on the analysis of the bands ascribed to the stretching vibration of ester C=O bonds in the polar head region of the membrane. The position of this band is strongly influenced by the hydration of the membrane as well as the packing of the lipid acyl chains and headgroups.^51,52^ The more fluid the membrane is, the greater the exposure of the C=O bond to the water environment, which is manifested by the shift of the corresponding band toward lower wavenumbers. For DMPC:DMPS membrane (Figure 7A), the complex band related to ester C=O bond vibration can be deconvoluted into the 3 sub-bands at ∼1742 cm^–1^, 1734 cm^–1^ representing poorly hydrated forms, and ∼1722 cm^–1^ corresponding to the fully hydrated form.

The contributions to this band are almost equally distributed between the component corresponding to strong hydration of the polar region at ∼1722 cm^–1^ and two components corresponding to dehydrated or poorly hydrated states at ∼1742 cm^–1^ and 1734 cm^–1^. For the deconvoluted spectra of the cancer model cell membrane after interaction with nonfunctionalized EVs (Figure 7B), some small shifts of the bands can be noticed. However, relevant information can be extracted based on the analysis of the relative bands’ intensities for different hydration states. There is a noticeable increase in the total area of bands corresponding to the weakly hydrated form at the expense of a significant decrease in the intensity of the band corresponding to the strongly hydrated form. Therefore, the number of strongly hydrated molecules has decreased, which may reflect an increase in the packing density of molecules in the membrane due to the transfer of lipids from EVs to the membrane. As a result, the distances between the polar heads decrease, leading to reduced hydration. In the case of the interaction between the DMPC/DMPS membrane and PTHTRWA-EVs (Figure 7C), the contribution of the band at ∼1720 cm^–1^ remains the same as for the intact bilayer, and thus, the overall contribution of bands corresponding to weak hydration is also similar to the reference system. However, there is a change in the proportion of intensity between the bands representing the dehydrated form and the poorly hydrated form in favor of the latter. The increased intensity of the band at ∼1736 cm^–1^ may indicate a slight increase in hydration (fewer molecules in the least hydrated state). This could be the result of a minor loosening of the membrane structure, possibly due to the partial transfer of lipid molecules from the membrane to the vesicles. A completely different substructure of the C=O band appears in the case of the DMPC:DMPS system interacting with EVs-PTHTRWA. In this case, a clear increase in the fraction of strongly hydrated molecules is observed, primarily at the expense of the band at ∼1736 cm^–1^, while the contribution from the weakest hydrogen-bonded groups remains comparable to the reference system. Interpreting this spectrum is rather complex, because an increase in hydration would imply a looser packing of molecules, which contradicts the interpretation based on υ(CH)_as_ band analysis. However, as suggested earlier, in this case, there may be a process of vesicle fusion, and the increased fraction of strongly hydrogen-bonded carbonyl groups could result from interactions with their counterparts in the polar head regions of the vesicles, as well as with anchored peptide chains on their surfaces.

**Figure 8:**
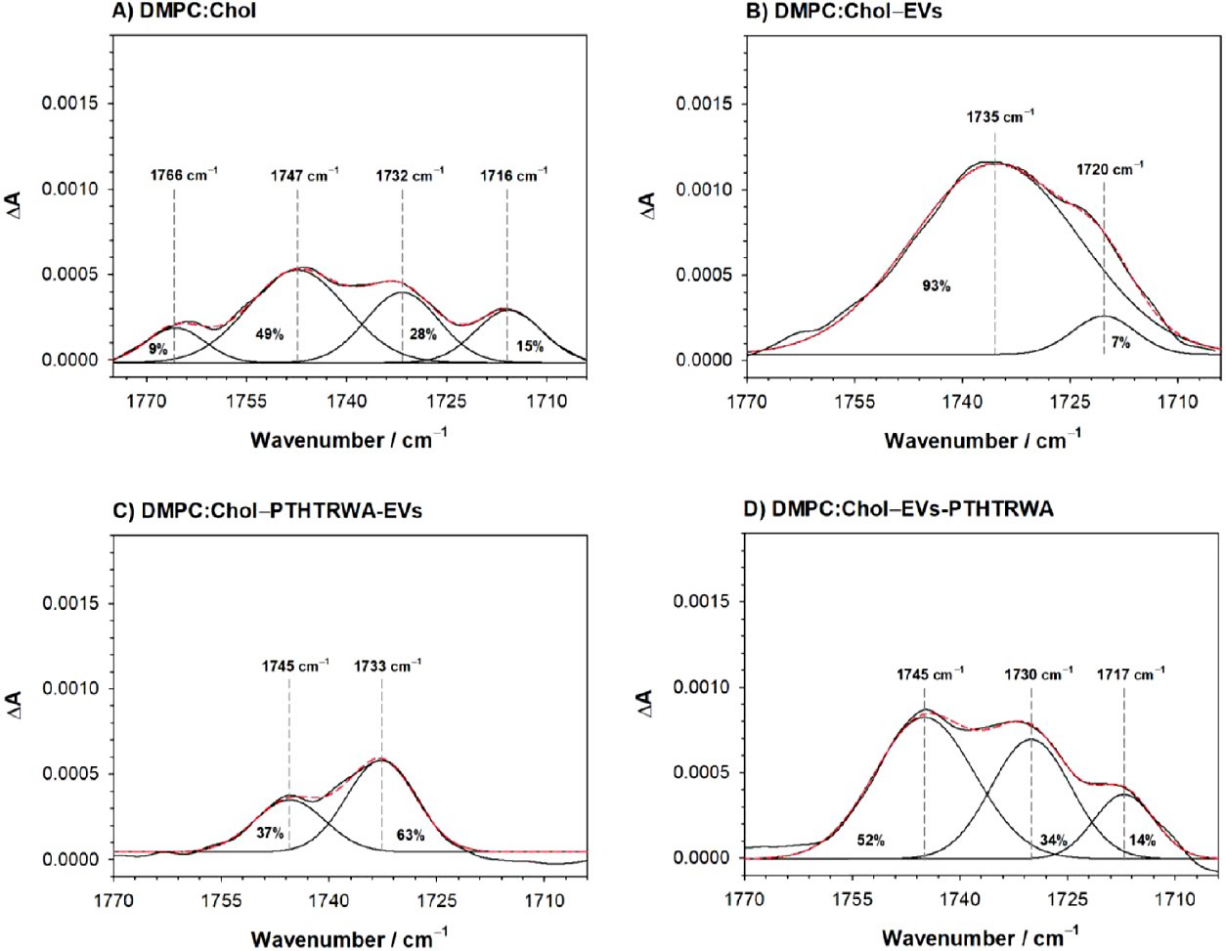
Deconvoluted spectra of the C=O bond for the membrane DMPC:Chol, which represents the normal cell membrane (A), and membrane after interaction with nonfunctionalized (B) and appropriately functionalized EVs (C and D).

A similar analysis was performed for the model of a DMPC/Chol membrane; see Figure 8. Compared to the previous model, an additional band is observed where the C=O bond is devoid of any internal interactions/additional hydrogen bonds. In addition, the well-hydrated band shifts toward lower wavenumbers (∼1716 cm^–1^, Figure 8A). Similar behavior was observed for the lipid membrane after the interaction with EVs-PTHTRWA (Figure 8D). However, the band presence at 1765 cm^–1^ is not observed in this case. Otherwise, the position and relative intensities of the bands remain similar, indicating rather limited influence of the EVs-PTHTRWA. Nevertheless, slightly increased overall contribution from the poorly hydrated and well-hydrated states may reflect a minor effect related to the partial transfer of the lipid material from the membrane to exosomes leading to slight loosening of molecular packing. The spectra for DMPC:Chol membrane with PTHTRWA-EVs are characterized by two bands (Figure 8C). One is located at 1747 cm^–1^, which is characteristic of a dehydrated C=O bond, and the second representing also poorly hydrated form is around 1733 cm^–1^. The contribution from the latter is significantly increased compared with the intact bilayer. This can be interpreted as the partial adsorption and attachment of EVs to the membrane surface. Their presence would reduce the availability of aqueous solution within the polar lipid heads of the membrane, hence the decrease in its hydration, but still, the polar heads would be involved in hydrogen bonding to some extent. Finally, for DMPC:Chol, the influence of the nonfunctionalized EVs leads to the more uniform hydration of the hydrophilic part of the membrane (Figure 8B). Of the four previously visible bands, the ν C=O band now consists mainly of one component located at 1735 cm^–1^ and a small signal around 1720 cm^–1^. In this case, a significant decrease in membrane hydration is observed, which is in line with the assumed increased packing density of lipids based on υ(CH)_as_ band analysis.

**Figure 9:**
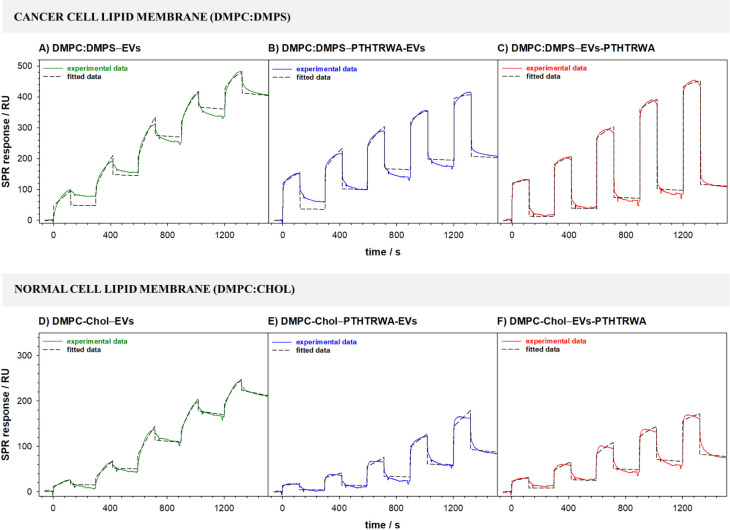
Sensorgrams recorded during the interactions of nonfunctionalized and appropriately functionalized EVs with cancer (A–C) and normal (D–F) cell lipid membranes. Experimental conditions: 0.01 M PBST-Gibco (pH 7.4), *C*_EVs_: 0.16–1.60 pM (1.0 × 10^8^–1.0 × 10^9^ particles·mL^–1^).

Studies of interactions occurring on the surface of the cell membrane exposed to EVs were also performed by using the SPR technique. The representative SPR response curves recorded during the binding process of nonfunctionalized and appropriately functionalized EVs to model cell membranes are shown in Figure 9. On the sensorgrams for different concentrations of EVs, two components related to the association and dissociation phase can be distinguished. During the association phase (the time during which the analyte flows through the measuring cell), the process of EVs binding to the cell membrane, modifying the chip surface, takes place. The rate of binding depends on the strength of the interaction and the rate at which EVs pass from the solution to the cell membrane. The binding of EVs to the membrane components results in an increase in the signal until equilibrium is reached (if that is achieved). The SPR response during the association phase should follow an exponential. Such a typical exponential shape was observed only for nonfunctionalized EVs with both normal and cancer cell membranes. In the case of the interaction of EVs decorated with heptapeptide, the initial binding rate is linear in the first few seconds (rapid growth) and then an exponential. The linear binding rate is a consequence of a too high amount of receptor on the chip surface. It should be stressed that the number of cell membrane interaction sites (receptors) with EVs is their intrinsic feature. Moreover, increasing the flow rate did not eliminate the initial linear increase. With the replacement of the EV solution by buffer solution (the dissociation phase), a decrease in the SPR signal intensity was observed resulting from the washing out of the EVs from the membrane surface. When in the final phase of dissociation the signal intensity reaches the baseline level (just before injection), no stable analyte–ligand complex (EVs-cell membrane) is formed. It is likely that in such a situation, the EVs interact with the cell membrane surface by adsorption without a subsequent step of cell membrane penetration, which is consistent with the ATR-IR results.

To estimate the kinetics parameters of the EVs-model, cell membrane interactions, such as association rate (*k*_a_), dissociation rate (*k*_d_), and equilibrium dissociation constant (*K*_D_), were evaluated by fitting a mathematical model (1:1 binding model) of the interaction to the experimental data. The dependencies ln(*R*_0_/*R*) = f(*t*), where *R*_0_ is the response level at the beginning of the postinjection phase for each EV concentration, plotted for all studied cases are linear. Thus, can be concluded that the chosen model correctly describes the interactions of nonfunctionalized and functionalized EVs with the components of model cell membranes.^53^ The estimated values of the kinetic parameters are provided in Table 1.

**Table 1:** Kinetic Parameters of Interactions between Non-Functionalized and Functionalized EVs and Model Cell Membranes Using a 1:1 Binding Reaction Model

	*k*_a_ [M^–1^·s^–1^]	*k*_d_ [s^–1^]	*K*_A_ [M^–1^]	*K*_D_ [M]
Cancer Cell Lipid Membrane DMPC:DMPS (7:3)				
nonfunctionalized EVs	1.84 × 10^6^	7.12 × 10^–3^	2.58 × 10^8^	3.87 × 10^–9^
PTHTRWA-EVs	7.63 × 10^4^	2.56 × 10^–6^	2.98 × 10^10^	3.36 × 10^–11^
EVs-PTHTRWA	5.16 × 10^4^	3.32 × 10^–4^	1.55 × 10^8^	6.43 × 10^–9^
Normal Cell Lipid Membrane DMPC:Chol (7:3)				
nonfunctionalized EVs	9.02 × 10^4^	2.18 × 10^–3^	4.13 × 10^7^	2.42 × 10^–8^
PTHTRWA-EVs	4.74 × 10^3^	3.52 × 10^–4^	1.34 × 10^7^	7.43 × 10^–8^
EVs-PTHTRWA	1.07 × 10^5^	1.81 × 10^–3^	5.92 × 10^7^	1.69 × 10^–8^

**Figure 10:**
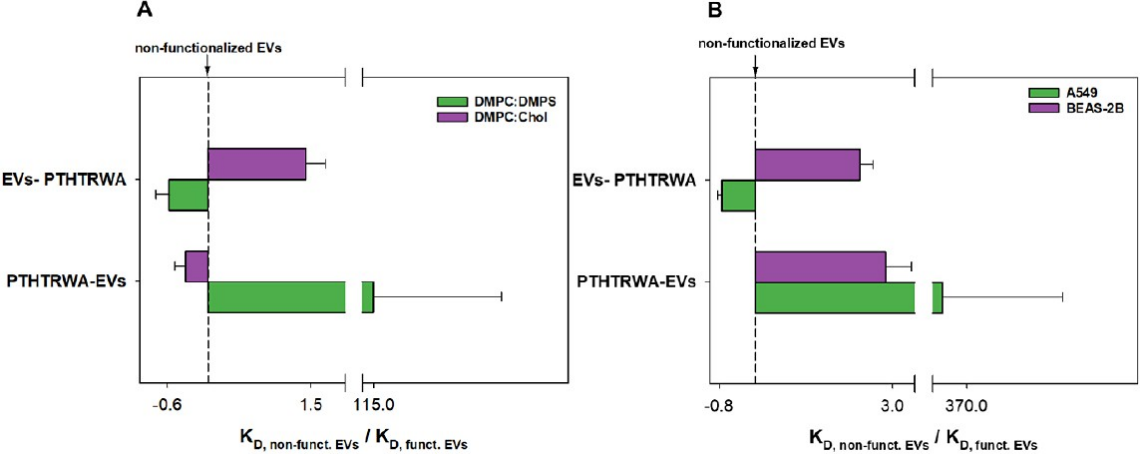
Ratio of *K*_D_ values for nonfunctionalized EVs and EVs functionalized with heptapeptide for the interaction with model membranes (A) and A549 and BEAS-2B cells (B).

Based on the *K*_D_ values obtained, it can be concluded that decorating EVs with a heptapeptide through its C-end significantly increases the affinity of EVs toward the cancer cell membrane (changing the value of the dissociation constant by 2 orders), see Figure 10.

**Figure 11:**
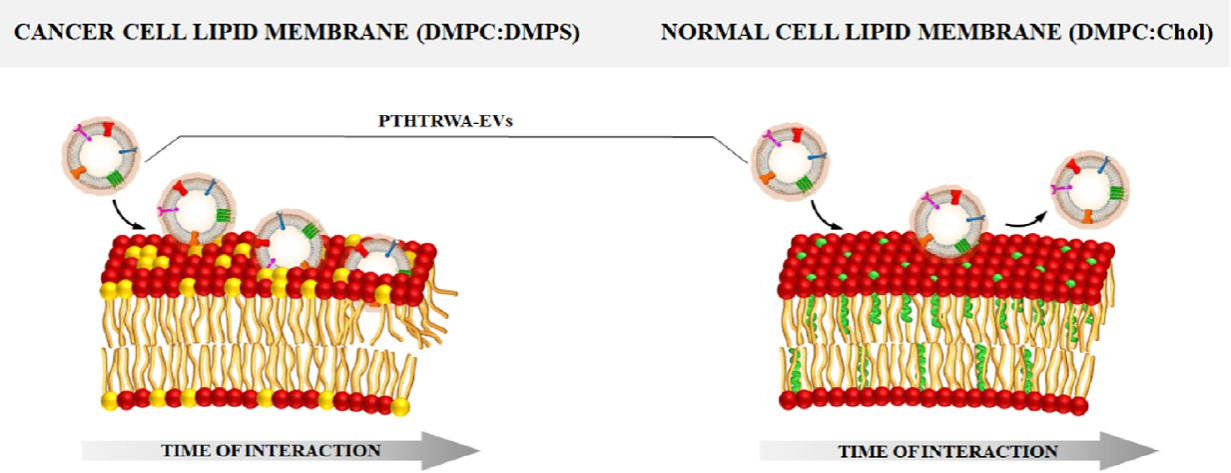
Scheme of the interaction of PTHTRWA-EVs with model cancer and a normal cell lipid membrane.

Anchoring of the targeting peptide on the surface of EVs through their N-end does not affect the strength of their interaction with the cancer cell membrane. Functionalization of the EV surface with the heptapeptide did not affect the affinity of the EVs for the normal model cell membrane. It is known that the association rate value higher than 10^5^ M^–1^·s^–1^ suggests the electrostatic type of interaction.^54^ Please note that such interactions were also supported based on MD studies. Taking into account the *k*_a_ values, it is clear that the contact of the functionalized EVs with the cell membrane leads to its rearrangement as well as changes in the hydration degree, which is schematically illustrated in Figure 11.

### 3.3 Affinity of Extracellular Vesicles Decorated with Targeting Heptapeptide toward Human Lung Cancer Cells and Tissues

**Figure 12:**
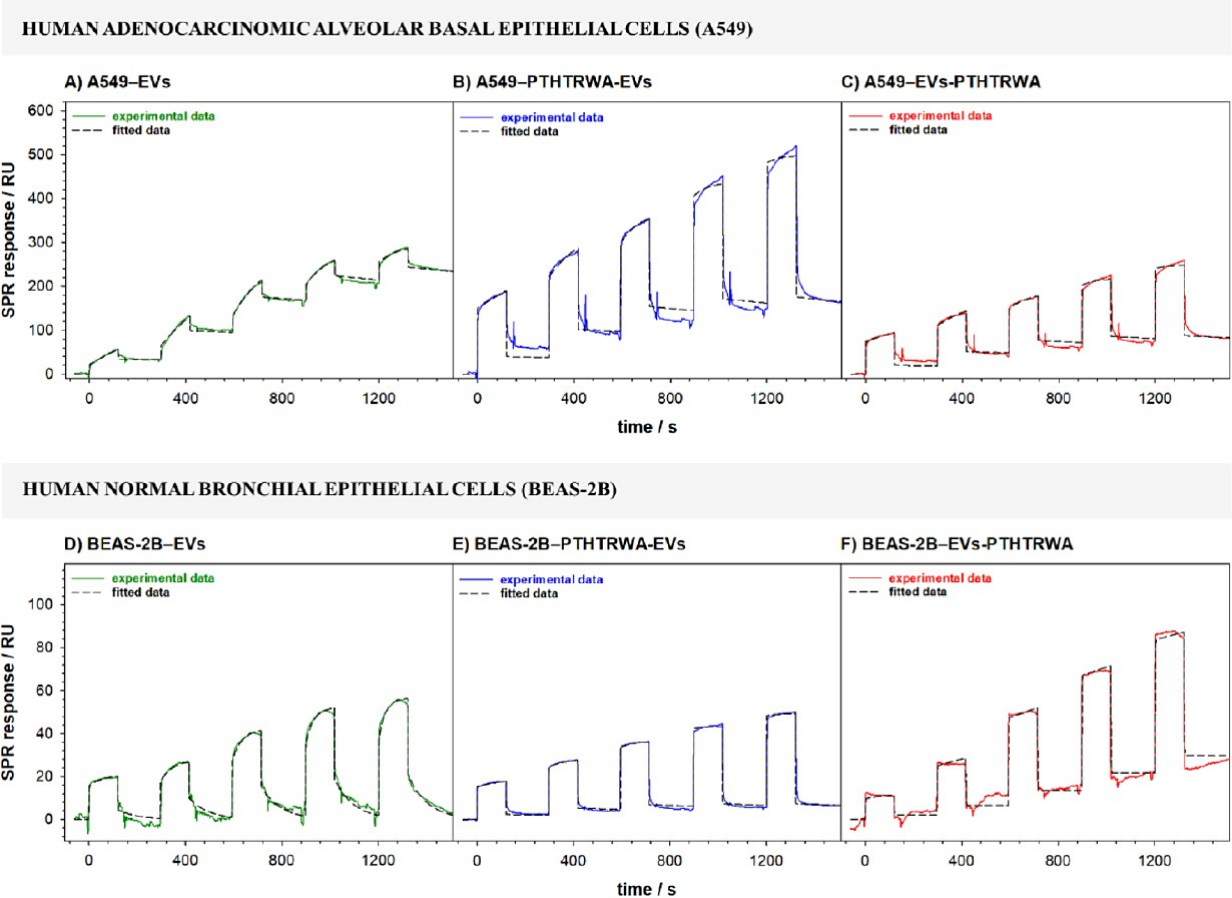
Sensorgrams recorded during the interactions of nonfunctionalized and appropriately functionalized EVs with human adenocarcinomic alveolar basal epithelial (A–C) and human normal bronchial epithelial (D–F) cells. Experimental conditions: 0.01 M PBST- Gibco (pH 7.4), *C*_EVs_: 0.16–1.60 pM (1.0 × 10^8^–1.0 × 10^9^ particles·mL^–1^).

The cell membrane is composed of lipids and proteins. Cancer cell membrane composition differs from the normal cell profile, moreover, it varies malignancy types.^55,56^ Furthermore, depending on the physiological condition, the cancer cell composition may fluctuate in time. Thus, lipids are not the only components of the cell membrane with which EVs functionalized with a self-navigating molecule can interact. To prove that extracellular vesicles decorated with a heptapeptide definitively increase the affinity of the system (EVs-heptapeptide) against only lung cancer cells, measurements using human adenocarcinomic alveolar basal epithelial cells were performed. In the control experiments, human normal bronchial epithelial cells were applied. The changes in the SPR signal intensity during exposure to the selected cells on nonfunctionalized and functionalized EVs are presented in Figure 12. Based on the recorded sensorgrams, it is evident that EVs functionalized with heptapeptide through its C-end (PTHTRWA-EVs) fully met the requirements of a model drug delivery system: they exhibit high affinity toward their dedicated A549 cancer cells (significant increase in SPR signal intensity compared to nonfunctionalized EVs) and permanently bind to the membrane of cancer cells (SPR signal intensity at the end of the dissociation phase is significantly higher than the response level at the beginning of the postinjection phase for each EV concentration), which facilitates the delivery of cargo (drug) to the cells. In contrast, the functionalization of EVs with a heptapeptide through its N-end (EVs-PTHTRWA) also improves their affinity for A549 cancer cells but they do not bind as firmly to the membrane of cancer cells as PTHTRWA-EVs. A control experiment performed with BEAS-2B cells proved that EVs-PTHTRWA shows a slightly higher affinity, some ability to interact, and not very stable binding with their membrane contrary to EVs and PTHTRWA-EVs, evidenced by the SPR signal intensity in the final phase of the dissociation process, which, unlike the signal obtained in the case of EVs and EVs-C, did not reach the baseline level (before EV injection). However, the interaction of nonfunctionalized and functionalized EVs with BEAS-2B cells is definitely weaker than with A549 cells.

A numerical description of the interactions of nonfunctionalized and heptapeptide-functionalized EVs with normal and cancer cells is presented in Table 2. A typical cell membrane contains various types of proteins in its structure, in addition to lipids. Proteins, due to their structure, depending on the pH of the environment, can change their surface charge. Thus, the main way the membrane proteins interact with EVs is through electrostatic interactions such as ion–ion, ion–dipole, or dipole–dipole. Such interaction types are found in computational studies. Taking into account the determined values of the association rate (*k*_a_ > 10^5^ M^–1^·s^–1^) of the process of EVs/EVs-heptapeptide association with A549 cancer cells, it can be concluded that the electrostatic nature of the interaction dominates. As a result of electrostatic attraction, EVs adsorb on the surface of the cell membrane and then penetrate its structure through respective receptors such as integrins. Our recent studies evidence α2β1 and α5β1 integrins on extracellular vesicles collected from human lung cancer cells.^57^ It is noteworthy that the interaction of A549 neoplastic cells with EVs decorated with a heptapeptide through its C-end is characterized by the highest value of the association constant. In contrast, the interaction of normal cells with EVs has mixed character, with a decidedly smaller value of the equilibrium constant of the association process.

**Table 2:** Kinetic Parameters of Interactions between Non-functionalized and Functionalized EVs with BEAS-2B and A549 Cells Using a 1:1 Binding Reaction Model

	*k*_a_ [M^–1^·s^–1^]	*k*_d_ [s^–1^]	*K*_A_ [M^–1^]	*K*_D_ [M]
Human Adenocarcinomic Alveolar Basal Epithelial Cells				
nonfunctionalized EVs	7.55 × 10^5^	2.32 × 10^–4^	3.25 × 10^9^	3.07 × 10^–10^
PTHTRWA-EVs	8.93 × 10^6^	7.55 × 10^–6^	1.8 × 10^12^	8.45 × 10^–13^
EVs-PTHTRWA	2.69 × 10^5^	1.13 × 10^–4^	1.46 × 10^8^	4.20 × 10^–10^
Human Normal Bronchial Epithelial Cells				
nonfunctionalized EVs	1.58 × 10^5^	1.25 × 10^–2^	1.26 × 10^7^	7.91 × 10^–8^
PTHTRWA-EVs	2.02 × 10^5^	5.59 × 10^–3^	3.61 × 10^7^	2.77 × 10^–8^
EVs-PTHTRWA	3.59 × 10^5^	1.23 × 10^–2^	2.91 × 10^7^	3.44 × 10^–8^

**Figure 13:**
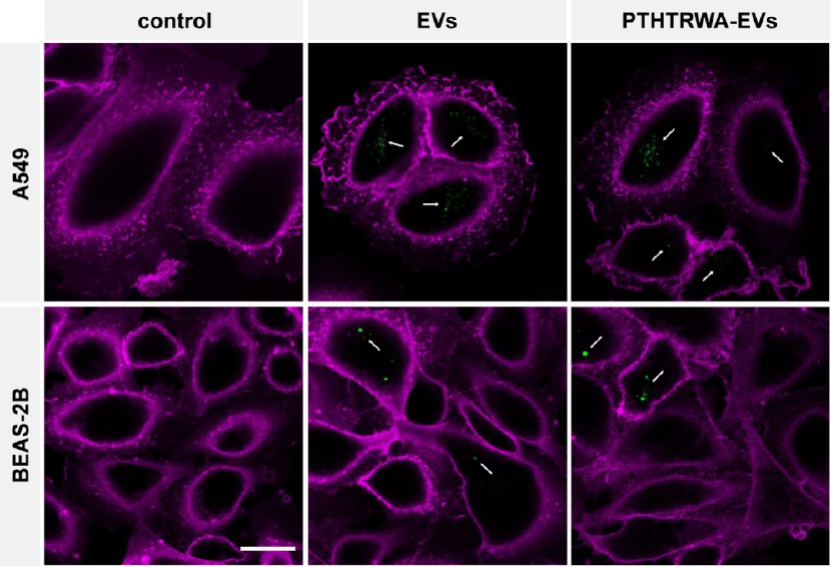
Uptake of EVs and PTHTRWA-EVs by A549 and BEAS-2B cells. Cells were exposed to DiOC18(3)-labeled EVs or PTHTRWA-EVs (both shown in green) for 2 h, fixed, and counterstained with wheat germ agglutinin-Alexa Fluor 647 (magenta) to visualize the cell membrane. EVs and PTHTRWA-EVs vesicles are internalized by A549 and Beas-2B cells (arrows). Controls (background staining control) were incubated with EV-free PBS that was treated like the EV solutions during the DiOC18(3) staining procedure. No residual dye was observed in the control-treated cells. Scalebar is 20 μm.

We performed the cellular uptake studies examining the uptake of pristine EVs and PTHTRWA-EVs by cancerous A549 and noncancerous BEAS-2B cells. To data, the cells were exposed to DiOC18(3)-labeled EVs or PTHTRWA-EVs and counterstained with wheat germ agglutinin-Alexa Fluor 647 (magenta) to visualize the cell membrane. We found that EVs and PTHTRWA-EVs are internalized by the A549 and BEAS-2B cells (Figure 13). The studies showed more pronounced uptakes for labeled PTHTRWA-EVs in cancerous A549 cells than noncancerous BEAS-2B cells, supporting other data of our studies that the as-bioengineered EVs could easily penetrate into lung cancer cells. Please note that extracellular vesicles released from some donor cells in the body could be up taken by donor cells with different mechanisms. Therefore, the cellular uptake of nonbioengineered EVs was also noted. One has to be emphasized that the lung cancer cells took up more extracellular vesicles than noncancer ones.

### 3.4 Transmembrane Integrin Targets for the PTHTRWA-EVs Construct

**Figure 14:**
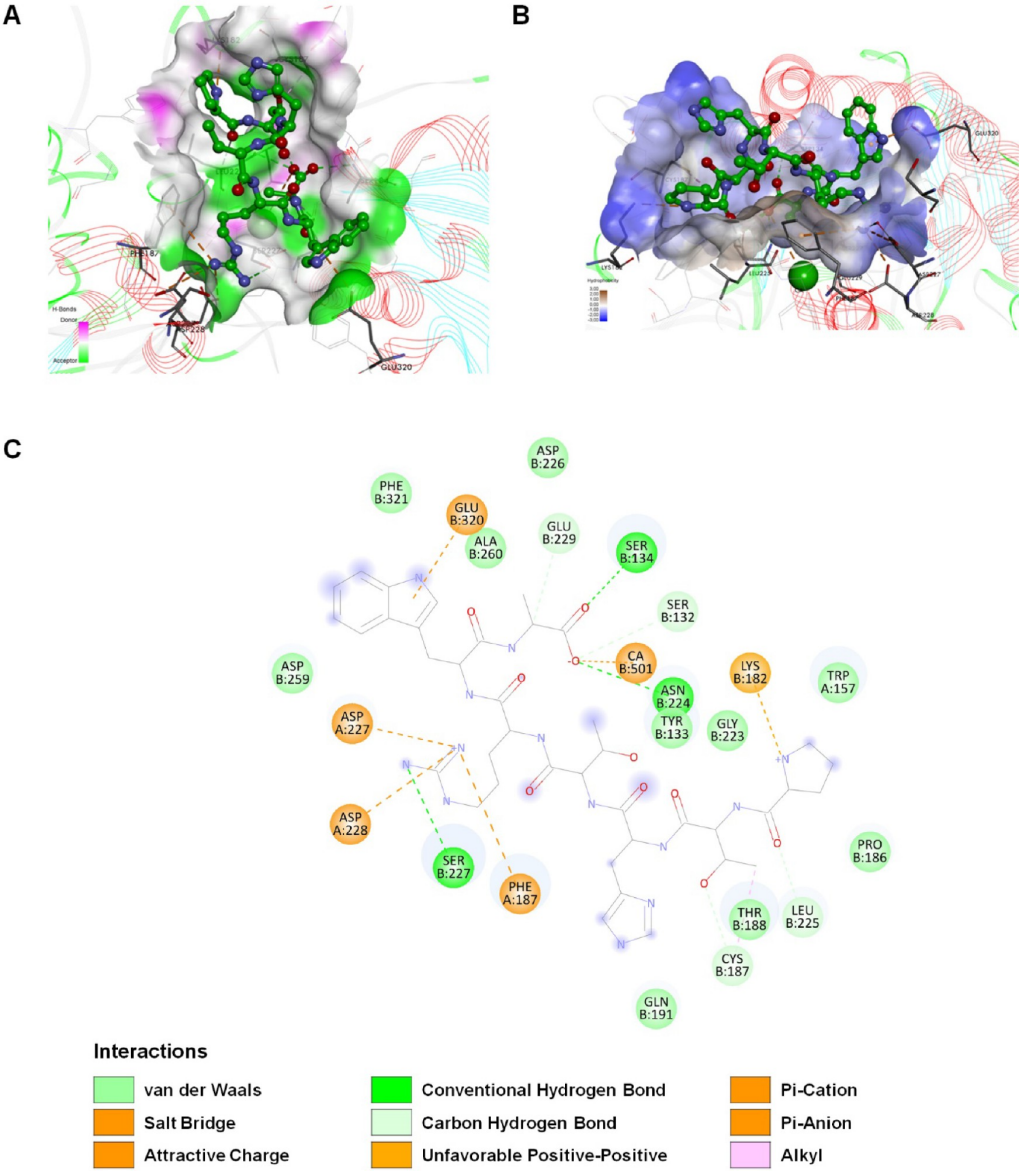
Binding mode of PTHTRWA to α5β1 integrin headpiece resulting from MD simulation. (A) Hydrogen bond surface of heptapeptide with α5β1. The hydrogen donor is presented using a pink color, while the hydrogen acceptor is presented as a lime surface. (B) The hydrophobic and hydrophilic amino acid residues surrounding the heptapeptide. Surface hydrophobicity is depicted using brown color—the hydrophobic and blue color—the lipophilic regions. (C) 2D view of all α5β1 residue interacting with the heptapeptide (residues involved in hydrogen bonds indicated as green and cyan circles; in hydrophobic interactions indicated as pink circles and electrostatic interactions indicated as orange circles).

Recent studies evidenced that integrins are highly expressed in different cancers, controlling cell proliferation and metastasis, and are responsible for promoting angiogenesis.^58^ To clarify the SPR results, the interaction of the heptapeptide PTHTRWA with α5β1 integrin, which is highly expressed in lung cancer cells as a transmembrane receptor,^59^ was investigated. First, molecular docking calculations were used to determine the optimal ligand position in the α5β1 integrin binding pocket. The crystal structure of 3VI4 was used for docking as a transmembrane receptor for PTHTRWA ligand. The CDOCKER program prosperously, placed the ligand into a receptor binding site, creating 30 positions for the compound. The position that showed the strongest interaction energies was chosen as the ligand starting structure for extensive molecular dynamic simulations. The predicted binding mode by CDOCKER is presented for peptide PTHTRWA in Figure S3. The results demonstrate that the analyzed peptide PTHTRWA was located at the exposed surface of a ditch-like top of the β1 subunit, which lacks the glycosylation sites.^60^ The binding site was consistent with the site in the crystal structure of the tripeptide RGD in integrin α5β1. Then, MD simulations were performed to study the dynamic binding process of a linear peptide PTHTRWA to the integrin α5β1. The binding free energy value calculated by the molecular mechanics Poisson–Boltzmann surface area method (MM-PBSA) was −35.87 kcal·mol^–1^, indicating the strong interactions of this integrin with PTHTRWA. The interaction between the PTHTRWA peptide and the integrin α5β1 during binding mainly involved conventional hydrogen bond, carbon–hydrogen bond, and electrostatic interaction due to the presence of amino acids such as Ser-132, Tyr-133, Ser-134, Trp-157, Lys-182, Pro-186, Cys-187, Phe-187 of α5, Thr-188, Gln-191, Gly-223, Asn-224, Leu-225, Asp-226, Ser-227, Asp-227 of α5, Asp-228 of α5, Glu-229, Asp-259, Ala-260, Glu-320, and Phe-321 in the vicinity of the ligand (Figure 14). Two types of electrostatic interactions were observed: interactions between residues and the metal ion, which contribute to the binding of the PTHTRWA to integrin α5β1, and interactions between residues, also known as salt bridges. The salt bridge was assessed by the distance between the donor and acceptor atoms. Hydrogen bonds and salt bridges are major contributors to the electrostatic interactions of proteins.^61^

The interaction site, hydrogen bond interaction space, interpolated charge, and hydrophobicity between the PTHTRWA and the integrin headpiece at the top of the β1 cavity are shown in Figure 14A,B. The hydrogen acceptor regions are displayed in green between PTHTRWA and integrin α5β1, while the hydrogen donor regions are shown in pink. The interaction space of the interpolated change between PTHTRWA and integrin α5β1 was from −0.1 to 0.1, a value of −0.1 represents a negative charge region of space, and a value of 0.1 represents a positive space region. The hydrophobicity between PTHTRWA and integrin α5β1 was from −3.0 to 3.0 such that the above interactive regions of space are shown to characterize the binding between heptapeptide and integrin. As shown in Figure 14C, the residues Ser-132, Ser-134, Lys-182, Cys-187, Asn-224, Leu-225, Ser-227, Glu-229, and Glu-320 on β1 have interacted with PTHTRWA. Furthermore, we observed the interactions of PTHTRWA with integrin residues derived from the α5-chain (Phe-187, Asp-228, and Asp-227) and with the Ca^2+^ ion in MIDAS. The ligand has a relationship with integrin β1 in conventional hydrogen bonds at the sites of Ser-132 (2.19 Å), Ser-134 (2.19 Å), Glu-229 (2.49 Å), Cys-187 (2.76 Å), Asn-224 (2.53 Å), Leu-225 (2.72 Å), and Ser-227 (2.19 Å). The position of peptide PTHTRWA allows for creating cation−π interaction between Arg^PTHTRWA^ and Phe-187 and between Pro^PTHTRWA^ and Lys-182 and anion−π interactions between Trp^PTHTRWA^ and Glu-320, where the distances were not over 4.0 Å. In addition, Asp-227 and Asp-228 have interacted with the peptide residue Arg^PTHTRWA^ via the salt bridge (2.49 and 2.64 Å, respectively). Simulation results also revealed that residue Ala^PTHTRWA^ binds to the Ca^2+^ ion at MIDAS, with a distance between them of 5.57 Å. Considering that the lung cell membrane has a negative charge, the as-designed peptide (PTHTRWA) has more likely access to the cell membrane via the electrostatic interaction, increasing the chance of contact with the transmembrane integrin α5β1receptor.

**Figure 15:**
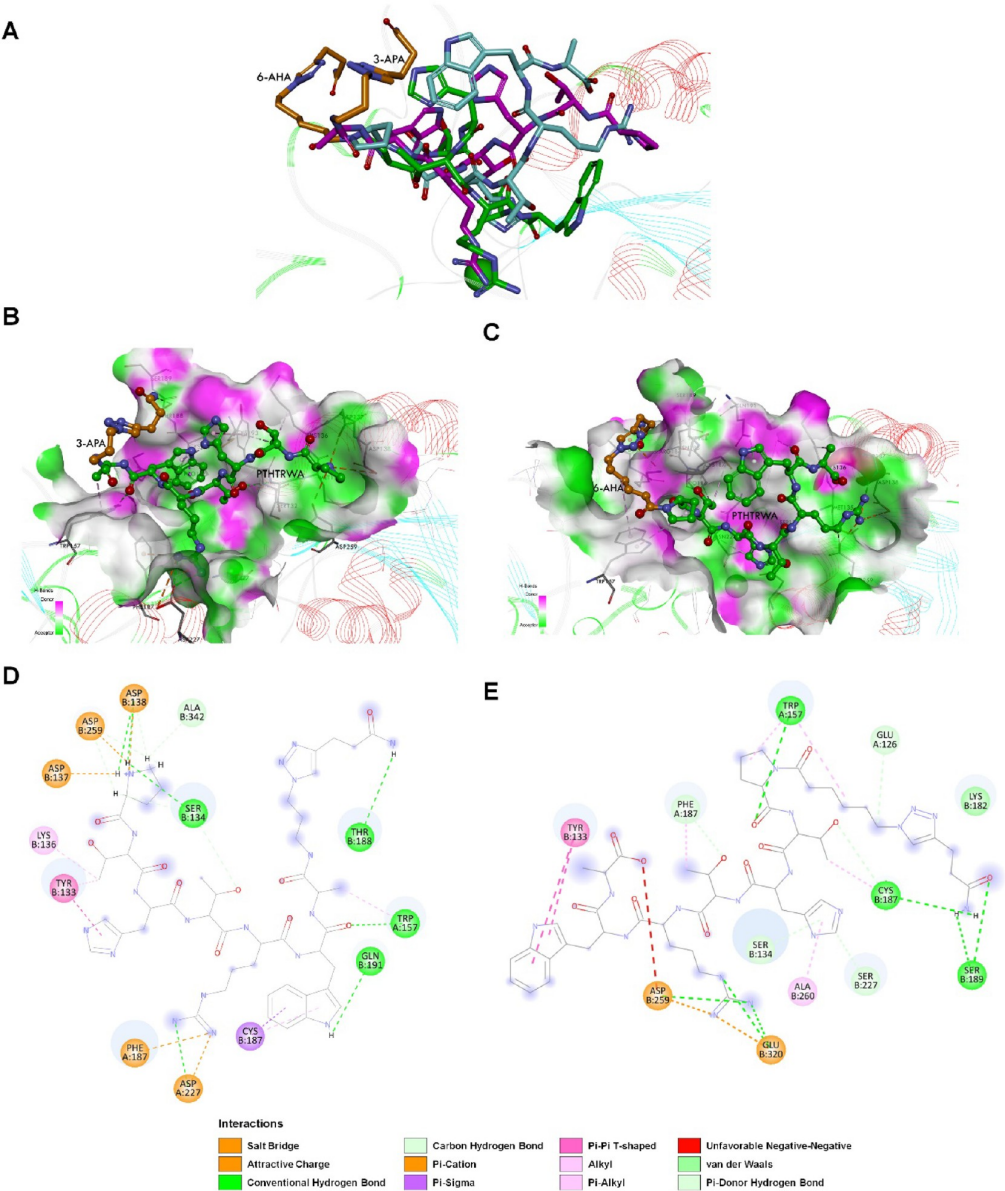
Predicted binding mode of N-terminated heptapeptide (6-AHA-PTHTRWA) and C-terminated heptapeptide (PTHTRWA-3-APA) to the α5β1 integrin headpiece resulting from MD simulation. (A) Superposition of compounds: PTHTRWA (C atoms shown in green), 6-AHA-PTHTRWA (C atoms shown in turquoise and orange), and PTHTRWA-3-APA (C atoms shown in pink and orange). (B) Hydrogen bond surface of PTHTRWA-3-APA with α5β1. (C) Hydrogen bond surface of 6-AHA-PTHTRWA with α5β1. The hydrogen donor is presented using the pink color, while the hydrogen acceptor is presented as a lime surface. (D) Molecular interactions between PTHTRWA-3-APA and α5β1. (E) Molecular interactions between 6-AHA-PTHTRWA and α5β1.

To rationalize the affinity of extracellular vesicles decorated with targeting PTHTRWA toward human lung cancer cells and tissues observed during the experimental studies, the effect of modifying the C- or N-terminal end of PTHTRWA on its binding to the α5β1 integrin receptor was additionally investigated. The research provided information about the nature of the sequences around PTHTRWA that are most favorable for α5β1 integrin binding. The MD resulting orientations of N-terminated heptapeptide (6-AHA-PTHTRWA) and C-terminated heptapeptide (PTHTRWA-3-APA), presented in Figure 15, define the geometric preferences of both compounds at the active site of the α5β1 integrin. As shown in Figure 15A–C, both compounds were located on the exposed surface of a ditch-like top of the β1, like a pure PTHTRWA. Noteworthy is the presence of the 3-azido-1-propanamine (3-APA) and 6-azido-hexanoic acid (6-AHA) groups in PTHTRWA, which determined the difference in binding modes of the heptapeptide, with both groups taking positions in the same location in the integrin pocket. The affinity of PTHTRWA-3-APA (−24.90 kcal·mol^–1^) is higher than that of 6-AHA-PTHTRWA (−20.10 kcal·mol^–1^) to integrin α5β1, which indicates that a more stable combination happens between the heptapeptide and integrin. The results demonstrate that when the PTHTRWA is attached via the C-terminal to exosomes, it will lead to a better affinity of such modified exosomes for cancer cells than exosomes with the PTHTRWA attached via the N-terminal. This suggests that the amino acid residues on the N-terminal side of the PTHTRWA appear to be more significant in integrin binding than the C-terminal ones.

As shown in Figure 14D, the residues Tyr-133, Ser-134, Lys-136, Asp-137, Asp-138, Cys-187, Thr-188, Gln-191, Asp-259, and Ala-342 on β1 have interacted with PTHTRWA-3-APA. Furthermore, we observed interactions of PTHTRWA-3-APA with integrin residues derived from the α5-chain (Trp-156, Phe-187, and Asp-227), similar to PTHTRWA. The ligand has a relationship with integrin β1 in conventional hydrogen bonds at the sites of Ser-134 (2.02 and 3.00 Å), Asp-138 (1.97, 2.16, and 2.30 Å), Thr-188 (2.15 Å), Gln-191 (2.15 Å), Asp-227 (1.92 Å), and Ala-342 (3.15 Å). The localization of PTHTRWA-3-APA allows for creating a cation−π interaction between Arg^PTHTRWA^ and Phe-187 (3.43 Å), and Asp-227 (4.77 Å), residues, and an π–σ interaction between Trp^PTHTRWA^ and Cys-187 (3.95 Å). In addition, Asp-137, Asp-138, and Asp-259 have interacted with peptide residue Pro^PTHTRWA^ via the salt bridge (3.24, 3.79, and 2.11 Å, respectively).

However, 6-AHA-PTHTRWA showed a more significant difference in interaction with α5β1 integrin compared to the other two compounds (PTHTRWA-3-APA and PTHTRWA), exhibiting lower affinity for the receptor. As shown in Figure 15E, the 6-AHA-PTHTRWA effectively filled the active site cavity of integrin α5β1 and was surrounded by the residues Glu-126 (α5), Tyr-133, Tyr-157 (α5), Lys-182, Cys-187, Phe-187 (α5), Ser-189, Leu-225, Ser-227, Asp-259, Ala-260, and Glu-320. It is worth noting that the interaction mechanism for the 6-AHA-PTHTRWA and the integrin was main through the hydrogen bonds and protein hydrophobic bonding force as shown in Figure 15E. The location of 6-AHA-PTHTRWA in the α5β1 integrin headpiece did not favor interactions with the peptide through the salt bridge. As seen in Figure 15E, 6-AHA-PTHTRWA is involved in unfavorable interaction with the Asp-259 residue of the chain for α5β1 integrin. The formation of unfavorable interactions in simulation results may indicate the presence of repulsive forces between ligand and target. Therefore, the generation of these unfavorable interactions can adversely influence the stability of the 6-AHA-PTHTRWA-α5β1 integrin complex in MD studies. The simulations reveal that the position of peptide 6-AHA-PTHTRWA allows for the creation of four strong hydrogen bonds between: Pro^AWRTHTP^ and Trp-157 (2.32 Å); Arg^AWRTHTP^ and Glu-320 (2.18 Å), and Asp-259 (2.14 and 2.39 Å). In addition, hydrophobic and electrostatic interactions may be observed with the main chain of Tyr-133 (5.10 and 6.06 Å), Trp-157 (4.16 and 5.03 Å), Phe-187 (α5) (4.80 Å), Asp-259 (4.78 Å), Ala-260 (5.36 Å), and Glu-320 (2.14 Å).

### 3.5 Magnetic Resonance Imaging of Mice Treated with SPIO-Loaded PTHTRWA-EVs

**Figure 16:**
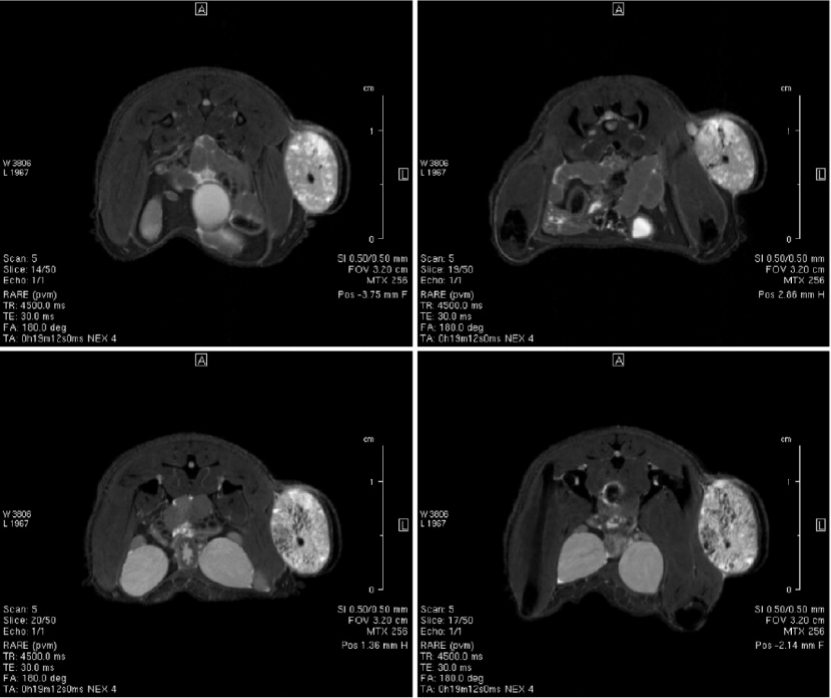
Representative T2-images of NUDE Balb/c mice bearing human lung A549 cancer. Mice were imaged before (left/top panel) and 1 h (right/top panel), 18 h (left/down panel), and 24 h (right/down panel) after intravenous injection (0.2 mg·mL^–1^) of SPIO-loaded PTHTRWA-EVs into the tail vain. In the tumor, numerous dark areas were appeared at 18 and 24 h postinjection. This was associated with decreased relaxation times (T2) in postinjected mice. MRI was performed using the Turbo RARE sequence (TR 4500 ms, TE 30 ms, FA 180.0 deg, TA 19 m 12 s, FOV 3.20 cm, MTX 256) in the axial plane. There is a clear blackening of the tumor areas accompanied by decreased signal intensities on T2-weighted images observed at the 18 and 24 h postinjection.

MRI is a diagnostic method used in both preclinical and clinical studies.^62^ This method is applied with or without contrast agents classified as positive or negative contrasts. Among negative contrast agents applied in MRI, iron oxide nanoparticles have been widely used also, playing a role as theranostics. These nanoparticles have been approved as contrast agents by regulatory bodies, including the Food and Drug Administration (FDA) and European Commission.^63^ To confirm the results collected based on the in vitro studies evidencing a high affinity of the PTHTRWA-EVs to the A549 cells, we loaded EVs with SPIO (see in the Supporting Information, Section 4) and intravenously injected the as-obtained SPIO-loaded PTHTRWA-EVs in normal saline to NUDE Balb/c mice bearing A549 cancer and subjected to MRI. Studies evidenced numerous darkness spots in some tumor areas targeted by SPIO-loaded PTHTRWA-EVs, which are accompanied in diminished signal intensities on T2-weighted images after 18 and 24 h postinjection (Figure 16). No such changes were observed on T2-weighted images in the tumor of the preinjected mice. This was accompanied by decreased relaxation times (T2) in the tumor ranging from 112.5 ± 3.5 ms (before injection) and 101 ± 2.8 (ms), 81.2 ± 2.5 (ms), and 77.8 ± 2.7 (ms) at 2-, 18-, and 24 h postinjection, respectively.

### 3.6 Preclinical Safety Studies

**Figure 17:**
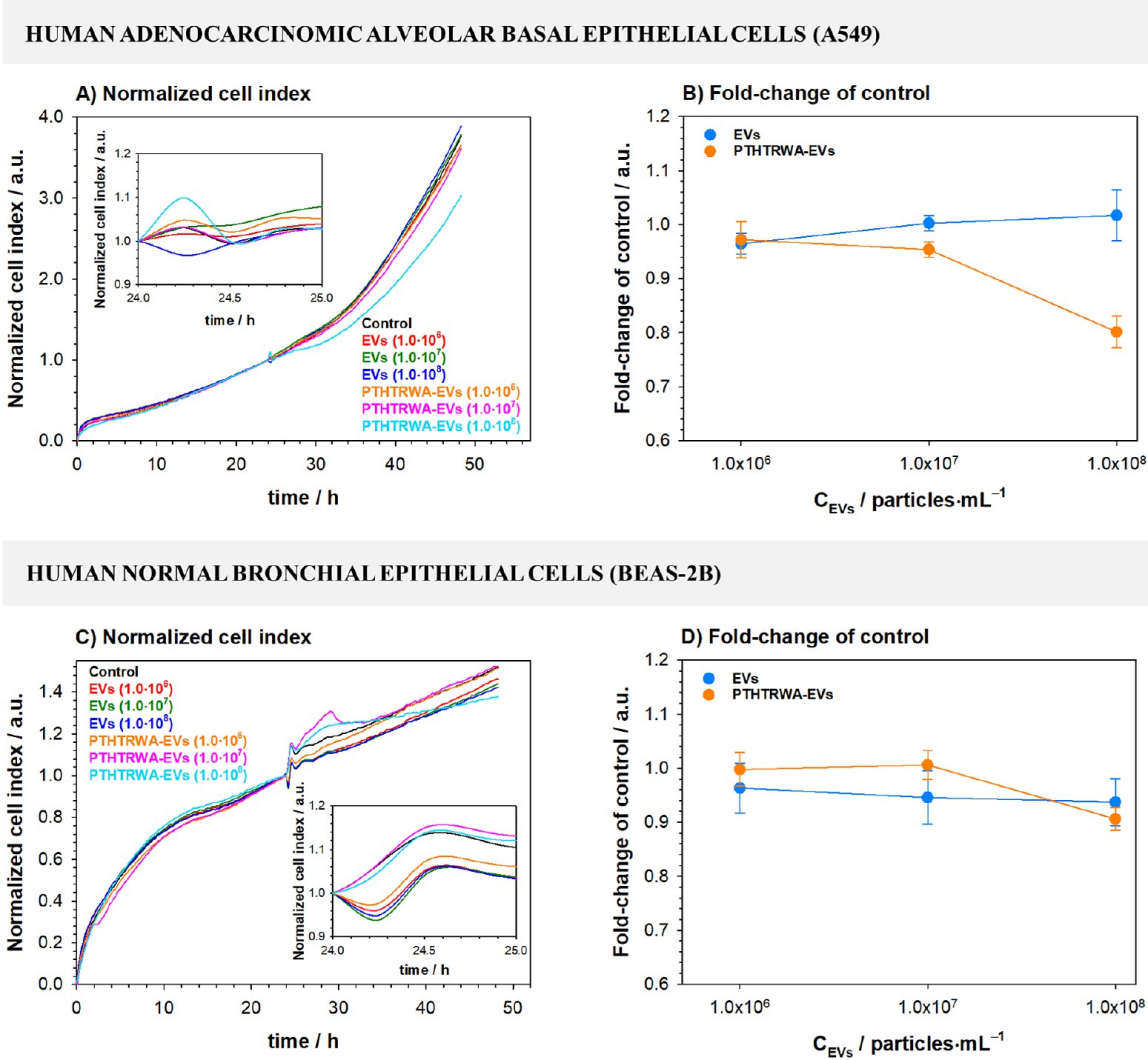
Effect of extracellular vesicles derived from lung cancer cells on lung cancerous and noncancerous cell growth and function assessed by electric cell–substrate impedance sensing. A549 cells (A, B), BEAS-2B cells (C, D). EVs – pristine extracellular vesicles; PTHTRWA-EVs – bioengineered extracellular vesicles. Cell index was normalized to the baseline control. EVs and PTHTRWA-EVs are expressed as particles·mL^–1^ unit.

ECSIS was used to assess the impact of the pristine EVs and bioengineered PTHTRWA-EVs at different concentrations on proliferation and viability of A549 and BEAS-2B cells and endothelial barrier integrity of a monolayer of HULEC-5a. Studies evidenced that A549 cells treated with the pristine EVs at different concentrations showed growth profiles (cell index vs time) that closely resembled the growth pattern of the negative control (Figure 17A). Consequently, no deleterious effect was observed by the end of the treatment (24 h, fold-change vs control). Treatment with peptide functionalized EVs at the highest concentration tested (1.0 × 10^8^ EV particles·mL^–1^) did cause a sudden increase of impedance upon the addition of these EVs (Figure 17A). This effect was followed by a fast drop and a subsequent gradual increase of impedance over time. Yet, the curve growth rate was slower than control, which resulted in slight, but significant, reduced cell growth at the end of the treatment time (24 h, fold-change vs control). No such effects were observed for the lower EV concentrations studied (Figure 17B). Treatment of BEAS-2B cells with nonfunctionalized EVs resulted in an initial drop of impedance, suggesting initial disruption of cell–cell and/or cell–substrate contacts caused by the presence of EVs, followed by continuous increase of impedance over time with no deleterious effect at the end of the treatment (Figure 17C). Growth profiles of BEAS-2B cells treated with bioengineered EVs at intermediate and highest concentration showed distinct features; a transient initial peak upon addition of extracellular vesicles followed by a slight drop of impedance and then sustained impedance growth at levels higher than control. This impedance growth peaked for treatment with the intermediate concentration and reached a plateau for the highest concentration at around 6 h after the addition of extracellular vesicles. This may suggest EV-mediated activation of cell surface receptors that leads to a transient increased cell–cell and/or cell–substrate contact strength. Yet, no statistically significant differences were observed when compared to control at the end of treatment (24 h, fold-change vs control) (Figure 17D).

Next, we studied the effect of the EVs on the endothelial barrier integrity. For this purpose, HULEC-5a was cultured at high cell density to let them form an endothelial monolayer. Immunostaining of the endothelial monolayer demonstrated localization of adherent and tight junction markers vascular endothelial (VE)-cadherin, zonula occludens-1 (ZO-1), and Claudin-5 at cell–cell contacts from day 3 of culture (see Figure S4). Thus, the culture conditions were deemed to be suitable for the formation of endothelial barriers by HULEC-5a. Afterward, we monitored the effect of EVs on endothelial barrier integrity via ECSIS. Treatment with thrombin, a serine protease that induces reversible disruption of endothelial barriers, caused a sharp drop of impedance within 15 min, which recovered back to baseline control level within 2 h. Treatment with pristine EVs and bioengineered EVs caused a moderate drop in impedance, especially for the intermediate and the highest concentrations of bioengineered EVs, that recovered to baseline control within 1 h. These results indicate that the EVs may affect endothelial barrier integrity by disrupting cell–cell contacts, but the effect is moderate and transient (Figure S5). One has to be noted that the cytotoxic and genotoxic studies based on Alamar Blue and Comet assays did not evidence any adverse effects of both pristine EVs and bioengineered PTHTRWA-EVs (see Figure S6).

## 4 Conclusions

The natural composition of extracellular vesicles has made these lipid-membrane-enclosed nanoparticles an attractive alternative to drug delivery systems and an innovative tool in personalized cancer therapy. Their specific features, such as high delivery efficiency, long half-life in circulation, intrinsic homing ability, biocompatibility, and low toxicity, set them apart from other drug carriers. EVs are excellent therapeutic shuttles also because of their origin, making them essentially invisible and unrecognized by the body, with regard to their relevance to physiological and pathological processes.

To improve the efficiency and selectivity of the interaction of EVs derived from human lung cancer cells (A549) with the target lesion site (human lung cancer cells), the surface of EVs was functionalized with the heptapeptide in two configurations through the C- or N-terminus of the peptide PTHTRWA. The introduction of the heptapeptide to the surface of EVs was confirmed by NMR, TEM, DLS, and ZP methods. In addition, it was proven that the EV bioengineering process does not cause aggregation of EVs. Studies conducted using the lipid membrane model mimicking healthy and lung cancer cell membranes and studies with human normal bronchial epithelial cells and human adenocarcinomic alveolar basal epithelial cells proved that EVs functionalized with the heptapeptide (PTHTRWA-EVs) through its C-terminus have a significantly higher affinity versus A549 cells. In turn, anchoring of the targeting peptide on the surface of EVs through its N-end (EVs-PTHTRWA) does not affect the strength of their interaction with the cancer cell membrane. Moreover, we proved that nonfunctionalized and appropriately functionalized EVs interact with the healthy cell membrane surface by adsorption without a subsequent step of cell membrane penetration. Only extracellular vesicles modified with heptapeptide through its C-end targeted and crossed only the cancer cell membrane barrier. This approach can reduce damage to the body’s healthy cells, which is an important issue for effective cancer treatment. In addition, heptapeptide-conjugated EVs may be a potential tool to reduce drug resistance and inhibit the proliferation of cancerous tumors and their metastasis to other organs. Preclinical MRI studies clearly evidenced that the SPIO-loaded PTHTRWA-EVs intravenously dosed in normal saline to NUDE Balb/c mice bearing human lung A549 cancer successfully reached the tumor within the first 24 h. Electric cell–substrate impedance sensing studies did not evidence any adverse effects for pristine EVs, but the studies showed some transient effects of peptide-functionalized EVs on both cancerous and noncancerous cells. Our results indicate that the EVs may affect endothelial barrier integrity, but the effect is moderate and transient. Note that the basic cytotoxic and genotoxic assays did not evidence any adverse effects of the pristine and as-bioengineered PTHTRWA-EVs. Therefore, it seems reasonable to conclude that the extracellular vesicles bioengineered with PTHTRWA peptide are good candidates for advanced therapy medicinal products and have great potential for further studies toward their medical applications in personalized lung cancer treatment.

## Data Availability

The data that support the findings of this study are available from the corresponding author upon reasonable request.

## Supporting Information Available

Heptapeptide H_2_N-Pro-Thr-His-Thr-Arg-Trp-Ala-OH synthesis; GC–MS studies (technical data and analysis); extracellular vesicles’ isolation and identification; MD in silico studies (data set preparation); interaction of heptapeptide with integrin α5β1 (MD studies); interaction of heptapeptide with integrin α5β (binding free energy calculations); preclinical safety in vitro studies (electric cell–substrate impedance sensing, immunostaining of endothelial barrier markers, Alamar Blue assay, genotoxicity by Comet assay); electroporation of superparamagnetic iron oxide nanoparticles into EVs (PDF)
