# Supplementary material for: Surface-Bioengineered Extracellular Vesicles Seeking Molecular Biotargets in Lung Cancer Cells
Source: ACS Appl Mater Interfaces. 2024 Jun 13;16(25):31997–2016. doi: 10.1021/acsami.4c04265 (PMC11212023; doi:10.1021/acsami.4c04265)
Supplement: Supplementary file 1 — am4c04265_si_001.pdf [file am4c04265_si_001.pdf]

Supporting Information

Surface-bioengineered extracellular vesicles seeking molecular biotargets in lung cancer cells

Agata Kowalczyk[1], Damian Dziubak[1,2], Artur Kasprzak[3], Kamil Sobczak[2], Monika Ruzycka-Ayoush[4], Magdalena Bamburowicz-Klimkowska[4], Sławomir Sęk[1,2], Ivan Rios-Mondragon[5], Teresa Żołek[6], Elise Runden-Pran[7], Sergey Shaposhnikov[8], Mihaela Roxana Cimpan[5], Maria Dusinska[7], Ireneusz P. Grudzinski[4,*], Anna M. Nowicka[1,*]

[1]Faculty of Chemistry, University of Warsaw, Pasteura Str. 1, PL-02-093 Warsaw, Poland

[2]Faculty of Chemistry, Biological and Chemical Research Centre, University of Warsaw, Żwirki i Wigury 101 Street, PL-02-089, Warsaw, Poland

[3]Faculty of Chemistry, Warsaw University of Technology, Noakowskiego Str. 3, 00-664 Warsaw, Poland

[4]Department of Toxicology and Food Science, Faculty of Pharmacy, Medical University of Warsaw, Banacha Str. 1, PL-02-097 Warsaw, Poland

[5]Biomaterials - Department for Clinical Dentistry, University of Bergen, Årstadveien 19, Bergen 5009, Norway

[6]Department of Organic and Physical Chemistry, Faculty of Pharmacy, Medical University of Warsaw, Banacha Str. 1, PL-02-097 Warsaw, Poland

[7]Health Effects Laboratory, Department of Environmental Chemistry, Norwegian Institute for Air Research, 2007 Kjeller, Norway

[8]Norgenotech AS, Gaustadalleen Str. 21, Oslo 0349, Norway

**Corresponding author:* ireneusz.grudzinski@wum.edu.pl (I.P.G.), anowicka@chem.uw.edu.pl (A.M.N.)

1. Heptapeptide H_2N-Pro-Thr-His-Thr-Arg-Trp-Ala-OH synthesis

The heptapeptide H_2N-Pro-Thr-His-Thr-Arg-Trp-Ala-OH was synthesized in 9 steps employing the solid phase peptide synthesis (SSPS) methodology.[1–3] The designed protocol is described below.

Step 1: Cesium salt of Boc-Ala-OH was synthesized following the literature protocol.[4] In brief, to a solution of Boc-Ala-OH (30 mmol) in EtOH (37.5 mL) and H_2O (12 mL), an aqueous solution (20 wt%) of cesium hydrogencarbonate was added until the pH of the mixture reached 7.0, under the electronic pH meter control. It was then stirred for 15 minutes at room temperature, concentrated in vacuo, and lyophilized for 48 hours to give Boc-Ala-OCs (quantitative yield) as a white solid.

Step 2: A suspension of Merrifield resin (4.1 g) and Boc-Ala-OCs (9.6 mmol) in DMF (30 mL) was stirred at 50 °C for 24 hours. It was then filtrated off using a 0,45 μm nylon membrane, washed with DMF, DMF/H_2O 9:1 *w/w*, and EtOH, and dried at room temperature for several days to give (Boc-Ala-O)-functionalized resin (5.8835 g) as a white solid.

Step 3: A suspension of (Boc-Ala-O)-functionalized resin (5.0 g) in 1.0 M HCl/AcOH (36 mL) was shaken (550 rpm) at room temperature for 40 minutes. It was then filtrated off using a Schott® separation funnel (porosity: G2). The wet resin was suspended in 1.0 M HCl/AcOH (24 mL) and shaken (550 rpm) at room temperature for 5 minutes, then filtrated off using a Schott® separation funnel (porosity: G2), washed with AcOH, EtOH and DMF. The wet resin was suspended in DMF (30 mL) and triethylamine (2 mL). It was shaken (550 rpm) at room temperature for 10 minutes, then filtrated off using a Schott® separation funnel (porosity: G2) and washed with DMF. The wet resin and a solution of Boc-Trp-OH (6 mmol) in DMF (10 mL) and dry DCM (30 mL) were stirred for 10 minutes at room temperature. A solution of DCC (6 mmol) in dry DCM (4 mL) was added, and the mixture was stirred for 24 hours at room temperature. It was then filtrated off using a nylon membrane, washed with DCM, DMF, and EtOH, and high vacuum for several hours to give (Boc-Trp-Ala-O)-functionalized resin (5.2011 g) as a white solid.

Step 4: A suspension of (Boc-Trp-Ala-O)-functionalized resin (5 g) in 1.0 M HCl/AcOH (36 mL) with the addition of DTE (0.5 wt%) was shaken (550 rpm) at room temperature for 40 minutes. It was then filtrated off using a Schott® separation funnel (porosity: G2). The wet resin was suspended in 1.0 M HCl/AcOH (12 mL) and shaken (550 rpm) at room temperature for 5 minutes, then filtrated off using a Schott® separation funnel (porosity: G2), washed with AcOH, EtOH and DMF. The wet resin was suspended in DMF (30 mL) and triethylamine (2

mL). It was shaken (550 rpm) at room temperature for 10 minutes, then filtrated off using a Schott® separation funnel (porosity: G2) and washed with DMF. The wet resin and a solution of Boc-Arg-OH (6 mmol) in DMF (30 mL) and dry DCM (30 mL) were stirred for 10 minutes at room temperature. A solution of DCC (6 mmol) in dry DCM (4 mL) was added, and the mixture was stirred for 24 hours at room temperature. It was then filtrated off using a nylon membrane, washed with DCM, DMF, and EtOH, and dried at high vacuum for several hours to give (Boc-Arg-Trp-Ala-O)-functionalized resin (5.2178 g) as a white solid.

Step 5: A suspension of (Boc-Arg-Trp-Ala-O)-functionalized resin (5 g) in 1.0 M HCl/AcOH (36 mL) with the addition of DTE (0.5 wt%) was shaken (550 rpm) at room temperature for 40 minutes. It was then filtrated off using a Schott® separation funnel (porosity: G2). The wet resin was suspended in 1.0 M HCl/AcOH (12 mL) and shaken (550 rpm) at room temperature for 5 minutes, then filtrated off using a Schott® separation funnel (porosity: G2), washed with AcOH, EtOH and DMF. The wet resin was suspended in DMF (30 mL) and triethylamine (2 mL). It was shaken (550 rpm) at room temperature for 10 minutes, then filtrated off using a Schott® separation funnel (porosity: G2) and washed with DMF. The wet resin and a solution of Boc-Thr-OH (6 mmol) in DMF (30 mL) and dry DCM (10 mL) were stirred for 10 minutes at room temperature. A solution of DCC (6 mmol) in dry DCM (4 mL) was added, and the mixture was stirred for 24 hours at room temperature. It was then filtrated off using a nylon membrane, washed with DCM, DMF, and EtOH, and dried at high vacuum for several hours to give (Boc-Thr-Arg-Trp-Ala-O)-functionalized resin (5.3891 g) as a white solid.

Step 6: A suspension of (Boc-Thr-Arg-Trp-Ala-O)-functionalized resin (5 g) in 1.0 M HCl/AcOH (36 mL) with the addition of DTE (0.5 wt%) was shaken (550 rpm) at room temperature for 40 minutes. It was then filtrated off using a Schott® separation funnel (porosity: G2). The wet resin was suspended in 1.0 M HCl/AcOH (12 mL) and shaken (550 rpm) at room temperature for 5 minutes, then filtrated off using a Schott® separation funnel (porosity: G2), washed with AcOH, EtOH and DMF. The wet resin was suspended in DMF (30 mL) and triethylamine (2 mL). It was shaken (550 rpm) at room temperature for 10 minutes, then filtrated off using a Schott® separation funnel (porosity: G2) and washed with DMF. The wet resin and a solution of Boc-His-OH (6 mmol) in DMF (60 mL) and dry DCM (20 mL) were stirred for 10 minutes at room temperature. A solution of DCC (6 mmol) in dry DCM (4 mL) was added, and the mixture was stirred for 24 hours at room temperature. It was then filtrated off using a nylon membrane, washed with DCM, DMF, and EtOH, and dried at high vacuum for several hours to give (Boc-His-Thr-Arg-Trp-Ala-O)-functionalized resin (5.2765 g) as a white solid.

Step 7: A suspension of (Boc-His-Thr-Arg-Trp-Ala-O)-functionalized resin (5 g) in 1.0 M HCl/AcOH (36 mL) with the addition of DTE (0.5 wt%) was shaken (550 rpm) at room temperature for 40 minutes. It was then filtrated off using a Schott® separation funnel (porosity: G2). The wet resin was suspended in 1.0 M HCl/AcOH (12 mL) and shaken (550 rpm) at room temperature for 5 minutes, then filtrated off using a Schott® separation funnel (porosity: G2), washed with AcOH, EtOH and DMF. The wet resin was suspended in DMF (30 mL) and triethylamine (2 mL). It was shaken (550 rpm) at room temperature for 10 minutes, then filtrated off using a Schott® separation funnel (porosity: G2) and washed with DMF. The wet resin and a solution of Boc-Thr-OH (6 mmol) in DMF (30 mL) and dry DCM (10 mL) were stirred for 10 minutes at room temperature. A solution of DCC (6 mmol) in dry DCM (4 mL) was added, and the mixture was stirred for 24 hours at room temperature. It was then filtrated off using a nylon membrane, washed with DCM, DMF, and EtOH, and dried at high vacuum for several hours to give (Boc-Thr-His-Thr-Arg-Trp-Ala-O)-functionalized resin (5.1979 g) as a white solid.

Step 8: A suspension of (Boc-Thr-His-Thr-Arg-Trp-Ala-O)-functionalized resin (5 g) in 1.0 M HCl/AcOH (36 mL) with the addition of DTE (0.5 wt%) was shaken (550 rpm) at room temperature for 40 minutes. It was then filtrated off using a Schott® separation funnel (porosity: G2). The wet resin was suspended in 1.0 M HCl/AcOH (12 mL) and shaken (550 rpm) at room temperature for 5 minutes, then filtrated off using a Schott® separation funnel (porosity: G2), washed with AcOH, EtOH and DMF. The wet resin was suspended in DMF (30 mL) and triethylamine (2 mL). It was shaken (550 rpm) at room temperature for 10 minutes, then filtrated off using a Schott® separation funnel (porosity: G2) and washed with DMF. The wet resin and a solution of Boc-Pro-OH (6 mmol) in DMF (5 mL) and dry DCM (30 mL) were stirred for 10 minutes at room temperature. A solution of DCC (6 mmol) in dry DCM (4 mL) was added, and the mixture was stirred for 24 hours at room temperature. It was then filtrated off using a nylon membrane, washed with DCM, DMF, and EtOH, and dried at high vacuum for several hours to give (Boc-Pro-Thr-His-Thr-Arg-Trp-Ala-O)-functionalized resin (5.2744 g) as a white solid.

Step 9: A suspension of (Boc-Pro-Thr-His-Thr-Arg-Trp-Ala-O)-functionalized resin (1.5 g) in 1.0 M HCl/AcOH (6 mL) with the addition of DTE (0.5 wt%) was shaken (550 rpm) at room temperature for 40 minutes. It was then filtrated off using a Schott® separation funnel (porosity: G2). The wet resin was suspended in 1.0 M HCl/AcOH (6 mL) and shaken (550 rpm) at room temperature for 5 minutes, then filtrated off using a Schott® separation funnel (porosity: G2), washed with AcOH, EtOH and DMF. The wet resin was suspended in DMF (6 mL) and triethylamine (1 mL). It was shaken (550 rpm) at room temperature for 10 minutes, then filtrated

off using a Schott® separation funnel (porosity: G2) and washed with DMF. The wet resin was transferred to a reaction flask and TFA (10 mL) with DTE (0.5 wt%) was added. HBr (48%; total 600 μL) was added in 4 portions every 5 minutes. The reaction mixture was stirred for 24 hours at room temperature. It was filtrated off using a Schott® separation funnel (porosity: G2) and washed with TFA (2 × 4 mL) with DTE (0.5 wt%). The as-obtained filtrate solution was added to MTBE (600 mL). The mixture was cooled at −28°C for 30 minutes, it was then filtrated off using a nylon membrane and washed thoroughly with MTBE. The as-obtained yellow-red oily solid was dissolved in dimethylsulfoxide (DMSO; 5 mL), filtrated off using a syringe filter (0.22 μm), and lyophilized for 48 hours to give H_2N-Pro-Thr-His-Thr-Arg-Trp-Ala-OH (266.1 mg) as a yellow-red oily solid. The as-obtained solid was stored in the dark under an argon atmosphere in a dry DMSO solution.

2. GC-MS studies

Technical data. To identify the amino acids, a sample of 1 mg of the synthesized heptapeptide was subjected to acid hydrolysis. The peptide was hydrolyzed using 6 M HCl at a temperature of 100 °C for 18 hours to release the amino acids, and 100 μL of the hydrolysate was obtained. After centrifugation for 15 minutes at 14,000 rpm, 50 μL of the supernatant was transferred to a vial and dried under a stream of nitrogen at 75 °C using a heated metal block. The resulting residue was dissolved in a mixture of 50 μL MSTFA and 50 μL pyridine and derivatized at 75 °C for 1 hour. The analysis was performed using a GC-MS-QP2010 gas chromatograph coupled with electron impact ionization (70 eV) and operated in scan mode with a scan range of m/z 33-600 (Shimadzu, Japan). The amino acid mixture was separated using a ZB-5MSi capillary column from Zebron, Phenomenex (0.25 mm i.d. × 30 m × 0.25 μm film thickness). The carrier gas (helium) flow rate was set to 1 $mL \cdot min^{-1}$. The initial oven temperature was 50 °C (held for 1 minute), then ramped up to 150 °C at a rate of 30 $°C \cdot min^{-1}$, and finally raised to 300 °C at 20 $°C \cdot min^{-1}$. The total run time for the analysis was 13 minutes. The injector temperature was maintained at 250 °C, and the injected volume was 1.0 μL with a split mode ratio of 20. The ion source and transfer line temperatures were set to 280 and 250 °C, respectively. The tentative identification of the amino acids was based on their mass spectra using the NIST05 version 3.2.4.05 library for comparison. A peak was considered identified when the experimental spectrum matched that in the library with a score above 90%.

GC-MS analysis. A representative peak intensity chromatogram of the hydrolyzed peptide is depicted in Figure. S1, peaks description is given in Table S1.

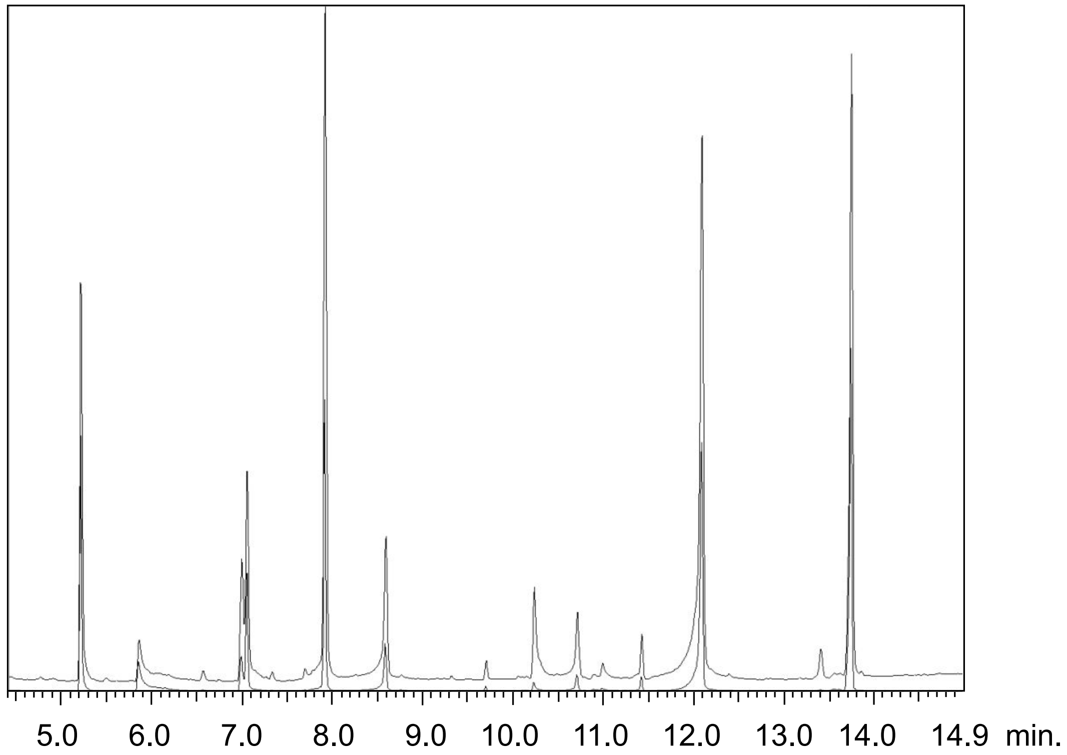

Figure. S1. Chromatogram of the silyl derivatives of amino acid after heptapeptide hydrolysis (see peaks description in Table S1).

Table S1. Composition of the heptapeptide based on silylated amino acids identified by GC-MS after peptide hydrolysis.

Retention time [min]	Amino acid	Identified derivative	Molecular mass of derivative
5.211	L-Alanine	*N*,*O*-Bis-(trimethylsilyl)alanine	233
5.850	L-Proline	*O*-trimethylsilyl proline	187
6.980	L-Threonine	Trimethylsilyl 2-amino-3-[(trimethylsilyl)oxy]butanoate	263
7.039	L-Proline	*N*,*O*-Bis-(trimethylsilyl)proline	259
7.910	L-Threonine	*N*,*O*,*O*-tris(trimethylsilyl)-L-threonine	335
8.580	L-Arginine	1-(trimethylsilyl)-3-[(trimethylsilyl)amino]-2-piperidinone	258
12.079	L-Histidine	*N*,*N'*,*O*-tris-(trimethylsilyl)histidine	371
13.731	L-Tryptophan	Trimethylsilyl 2-[(trimethylsilyl)amino]-3-[1-(trimethylsilyl)-1H-indol-3-yl]propanoate	420

3. Extracellular vesicles isolation and identification

Cell culture. Human adenocarcinomic alveolar basal epithelial cell line - A549 (ATCC CCL-185) and human bronchial epithelial cell line - BEAS-2B (ATCC CRL-9609) was obtained from American Type Culture Collection (ATCC, Manassas, VA, USA). The cells were cultivated under a 5% CO_2 atmosphere at 37 °C in a CO_2 incubator (Memmert, Schwabach, Germany). A549 cells were grown as an adherent monolayer in F-12K medium (Kaighn's modification of Ham's F-12 medium; Gibco, Paisley, supplemented with 10% fetal bovine serum (FBS; Gibco, Paisley, UK) and antibiotics (streptomycin, 50 $\mu g \cdot mL^{-1}$; amphotericin B, 1.25 $\mu g \cdot mL^{-1}$; gentamicin, 50 $\mu g \cdot mL^{-1}$; penicillin, 50 $\mu g \cdot mL^{-1}$) (Gibco, Paisley, UK). The BEAS-2B cells were cultured in serum-free LHC-9 medium with antibiotics (streptomycin, 50 $\mu g \cdot mL^{-1}$ and penicillin, 50 $\mu g \cdot mL^{-1}$). Both A549 and BEAS-2B cells were used to cover the SPR chip to investigate the activity of the functionalized EVs. A549 cells at passage 11, 12 and 13, and BEAS-2B cells at passage 2, 3 and 5 were used in the experiments. A549 cells were also used for EVs production. Prior to EVs isolation, the standard media was replaced with a 10% exosome-depleted FBS media (One Shot™ format, Gibco, Paisley, UK), and A549 cells were incubated for a further 3 days in T225 culturing flasks.

Extracellular vesicles isolation. The cell culture media was harvested from the cell cultures (A549) and centrifuged at 750×g at 4 °C for 15 min to remove the detached cells. Then, the collected media was centrifuged at 2000×g at 4 °C for 20 min to remove the microvesicles. The supernatant was then collected and filtered through 0.45-µm filters and subsequently spun in a Beckman Coulter Optima™ L-80XP Ultracentrifuge at 10,000×g at 4 °C for 45 min. with a Type SW 32 Ti rotor to remove the apoptotic bodies and cell debris. The supernatant was again recovered and filtered through 0.22-µm filters and ultracentrifuged at 100,000×g at 4 °C for 90 min to pellet the EVs. The supernatant was then carefully removed, and crude EVs-containing pellets were resuspended in an aliquot of PBS and pooled.

Extracellular vesicles identification. EVs used in this study were characterized in our recently published paper using such methods and techniques as transmission electron microscopy (TEM) with energy-disperse spectroscopy Super-X windowless drift detectors (EDX), Nanoparticle Tracking Analysis (NTA), Dynamic Light Scattering (DLS) and Western Blot. NTA and DLS were used to confirm the size of EVs, while Western Blot analysis served for the determination of canonical markers of EVs *i.e.* CD63 and CD81 and tumor susceptibility gene 101 (TSG101) also specific to EVs.[5]

4. Molecular dynamic in silico studies

Dataset preparation. The starting conformation of ligand PTHTRWA was constructed using the „Bild and Edit Protein" module of Discovery Studio v.21 interfaces BIOVIA.[6] The molecular structure of PTHTRWA was optimized using the density functional theory (DFT) with the B3LYP/6-311G (d,p) hybrid functional, as implemented in the Gaussian 16 program[7] (Figure. S2).

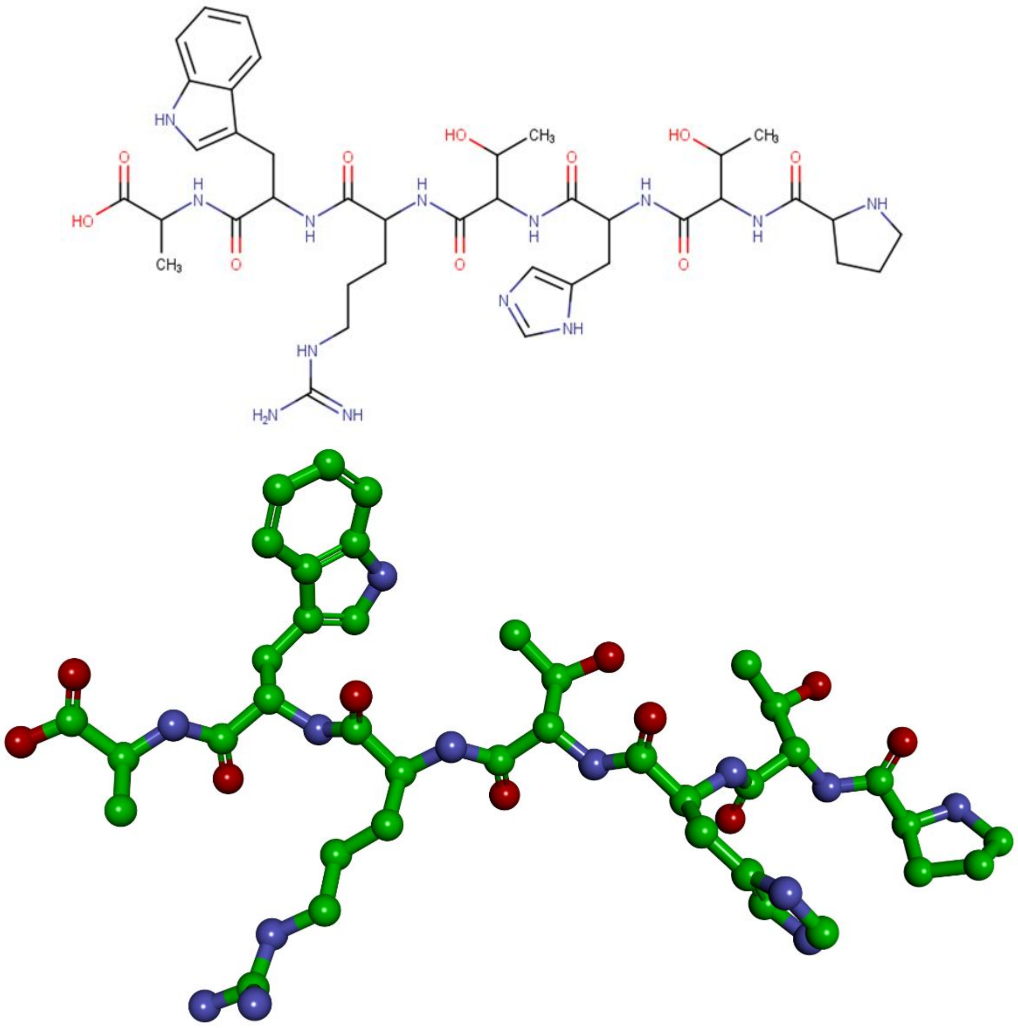

Figure. S2. The chemical structure of heptapeptide PTHTRWA (H_2N-Pro-Thr-His-Thr-Arg-Trp-Ala-OH) (**top**) and optimized molecular structure of PTHTRWA calculated by the DFT method at the B3LYP 6-311 (d,p) (**bottom**).

Partial atomic charges were assigned to the atoms using Breneman's model[8] reproducing the molecular electrostatic potential. The structural model of human integrin $\alpha5\beta1$ was obtained from the RCSB Protein Data Bank (3VI4, resolution of 2.9 Å[9]). The crystal structure of 3VI4 is the $\alpha5\beta1$ integrin headpiece bound by an allosteric inhibitory antibody (SG/19 Fab) and in the presence of an RGD (tripeptide ligand containing the Arg-Gly-Asp sequence) at physiological concentrations of Mg^{2+} and Ca^{2+} ions. The original ligand position and water

molecules of crystal structure were removed from integrin as an interaction site in the complex. For receptor preparation, the protein structure was added with explicit hydrogen atoms, and all the ionizable residues were set to a proton at pH 7.4. Molecular docking was performed using the CDOCKER algorithm implemented in the Discovery Studio 2021 software.[10] The binding site sphere of integrin was defined as the region that comes within a radius 15 Å from the position of the original ligand in the crystal structure of the receptor. The pose with the lowest CDOCKER interaction energy was selected as the best conformations for the binding with the active site of the integrin through hydrogen bonds and hydrophobic interactions. The predicted binding mode by CDOCKER is presented for peptide PTHTRWA in Figure. S3. The best pose predicted by CDOCKER was used as the starting point in the molecular dynamics (MD) simulation.

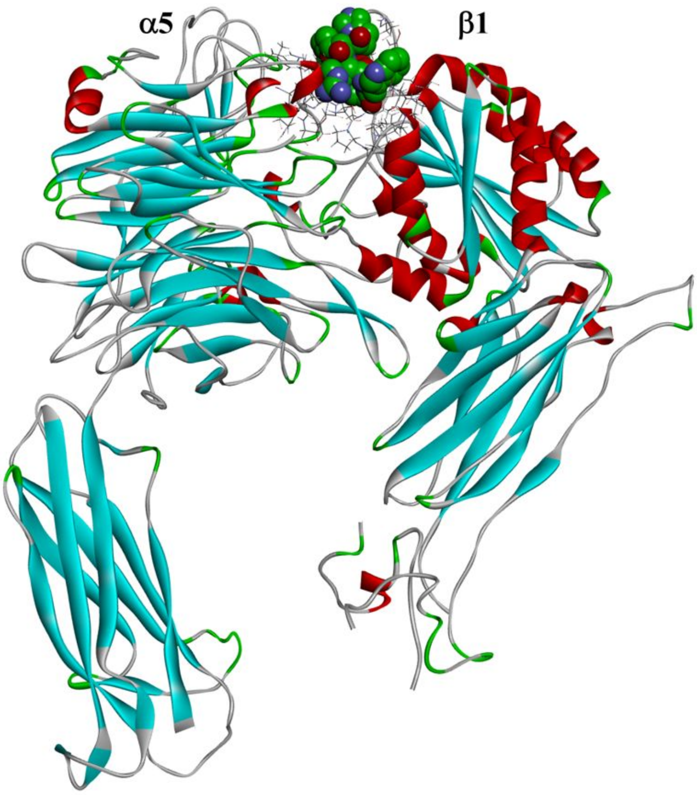

Figure. S3. Structure of the *α5β1* integrin headpiece (PDB: 3VI4) in the complex with heptapeptide after docking.

Interaction of heptapeptide with integrin *α5β1*: molecular dynamic (MD) studies. Energy minimizations and MD simulations were performed using the particle mesh Ewald (PME)

method for the correct treatment of electrostatic interactions. In the beginning, the minimization process was made using molecular mechanics (MM) in three stages. Firstly, the water molecules were optimized but the receptor was restrained; secondly, water molecules and the side chains of the receptor were optimized but the backbone of the receptor was restrained. The listed stages were performed by using two cycles of minimization: 5,000 steps of energy minimization, switching from steepest descent to conjugate gradient after 15,000 cycles. Third, the entire system was optimized without any constraints in 5,000 steps, starting with the steepest descent minimization, followed by conjugate gradient minimization after 10,000 cycles (until the RMS gradient of the structures dropped below 0.01 $kcal \cdot mol^{-1} \cdot Å^{-1}$). All restraints in this study were weak with a force constant of 2 $kcal \cdot mol^{-1} \cdot Å^{-2}$. The resulting minimized system was heated from 0 to 310 K for 200 ps. Prior to the production stage, the system was equilibrated by allowing it to evolve spontaneously until the average temperature and the structure remained stable and the total energy converged. The total number of steps to perform the dynamic simulation was 500 ps. Finally, an unbiased and unrestrained system went through production in a periodic boundary condition for 500 ns in NPT by Langevin thermostat. To analyze the correlated movement, we performed a 500 ns MD simulation for ligand-free integrin, the ligand alone, and integrin bound with a ligand. The quality of the integrin geometry and the structure folding reliability were determined by taking the stabilized structure from the trajectory of the system.

Interaction of heptapeptide with integrin $\alpha 5\beta 1$: binding free energy calculations. The atomic coordinates of the integrin and ligand were taken from a single trajectory of the MD simulation obtained using explicit water molecules. The last 10 ns of each MD trajectory was used to calculate MM/PBSA using a 50 ps interval, and 100 MD frames were extracted for each calculation. The components of each complex were minimized using the coupled gradient method for 10,000 steps, following 1000 steps of the steep descent algorithm and dielectric constant 4 for integrin and 80 for solvent, until the RMS gradient of the structure was less than 0.001 $kcal \cdot mol^{-1} \cdot Å^{-1}$. The obtained ΔG_{bind} considers the integrin fluctuations and the ligand

conformations, thus ensuring the correct location of the ligand within the binding pocket. The binding free energy integrin-ligand complex in a solvent system is stated as $\Delta G_{bind} = G_{complex} - G_{integrin} - G_{ligand}$, where $G_{complex}$ refers to the total free energy of the integrin-ligand complex and $G_{integrin}$ and G_{ligand} indicate the separated integrin and ligand in the solvent.

5. Preclinical safety *in vitro* studies

Electric cell-substrate impedance sensing (ECSIS). A549 and BEAS-2B cells were seeded at a density of 25000 cells·cm^{-2} and 50000 cells·cm^{-2}, respectively, in 16-well E-plates (Agilent, USA) containing a microelectrode array in the bottom of the wells (to note, E-plates were pre-coated with fibronectin/collagen-I/BSA for experiments with Beas2B cells). Then, E-plates were fitted in the real-time impedance analyser (xCELLigence RTCA, Agilent) and incubated for 24 h at 37 °C and 5% CO_2. Electrical impedance was monitored in real-time every 15 min at 10 kHz AC frequency. After the initial 24 h pre-exposure culture, EVs were added to the cell culture wells at concentrations $1.0{\cdot}10^6$, $1.0{\cdot}10^7$ and $1.0{\cdot}10^8$ EVs particles·mL^{-1} and the E-plates were fitted back in the impedance analyser and further cultured for 24 h. Negative controls were cells incubated in the presence of the dispersion media (PBS) at a concentration that corresponded to the volume of dispersion media contained in the highest EV concentration. In addition, cell-free wells were included to monitor the cell culture medium- and EV-derived impedance background. For CI analysis, background values were subtracted from the respective exposure conditions for each time-point. These corrected CI(t) values were normalised to the CI value at time-point 24 h, right before the beginning of EV-treatment, to take into account possible differences in number of cells seeded and uniformity of cell distribution onto the electrode array. Finally, the fold-change vs negative control was calculated at the end of the 24 h treatment period (48 h total incubation time).

To assess endothelial barrier integrity, HULEC-5a were seeded at a density of 75000 cells·cm^{-2} in 16-well E-plates and incubated for 3 days at 37 °C and 5% CO_2 to let cells form an endothelial monolayer. Electrical impedance was monitored in real-time every 15 min at 10 kHz AC and the cell culture medium was replaced every day. After the 3-day pre-exposure cultured, EVs were added to the cell culture wells at concentrations $1.0{\cdot}10^6$, $1.0{\cdot}10^7$, and $1.0{\cdot}10^8$ EVs particles·mL^{-1} and the E-plates further cultured for 24 h measuring impedance every 15 min. The negative control was prepared as mentioned above and the positive control was 5 U·mL^{-1} Thrombin in cell culture medium. CI values were background corrected and then normalized at time-point 72 h, right before the beginning of EV-treatment. Finally, we calculated the baseline normalized CI by subtracting the CI value of the negative control to each

treatment condition. All conditions were tested in duplicate wells and the data presented corresponds to the mean of three independent experimental repetitions.

Immunostaining of endothelial barrier markers. HULEC-5a were plated at a density of 75000 cells·cm^{-2} in 8-well IBIDI chamber slides (IBIDI, Germany) and cultured for 3 and 5 days at 37 °C and 5% CO_2 to let cells form an endothelial monolayer. After the culture time, wells were washed once with warm PBS containing Ca^{2+} and then cells were fixed with cold methanol for 10 min at -20 °C. Specimens were rinsed with PBS, blocked with a solution of 4% BSA / 4% FBS in PBS and then incubated 2 h at RT with 5 μg·mL^{-1} solutions of primary antibodies mouse anti-VE Cadherin (Cat. no.: 14-1449-82, ThermoFisher) and rabbit anti-ZO-1 (Cat. no.: 33-9100, ThermoFischer) or mouse anti-Claudin-5 (Cat. no.: 35-2500, ThermoFischer). The secondary antibodies used for detection of primary antibodies were goat anti-rabbit Alexa Fluor 546 (ThermoFischer) and goat anti-mouse Atto 488 (Sigma-Aldrich). After incubation with antibodies, cells were counterstained with DAPI and imaged using a confocal TCS SP8 fluorescence microscope (Leica Microsystems, Germany).

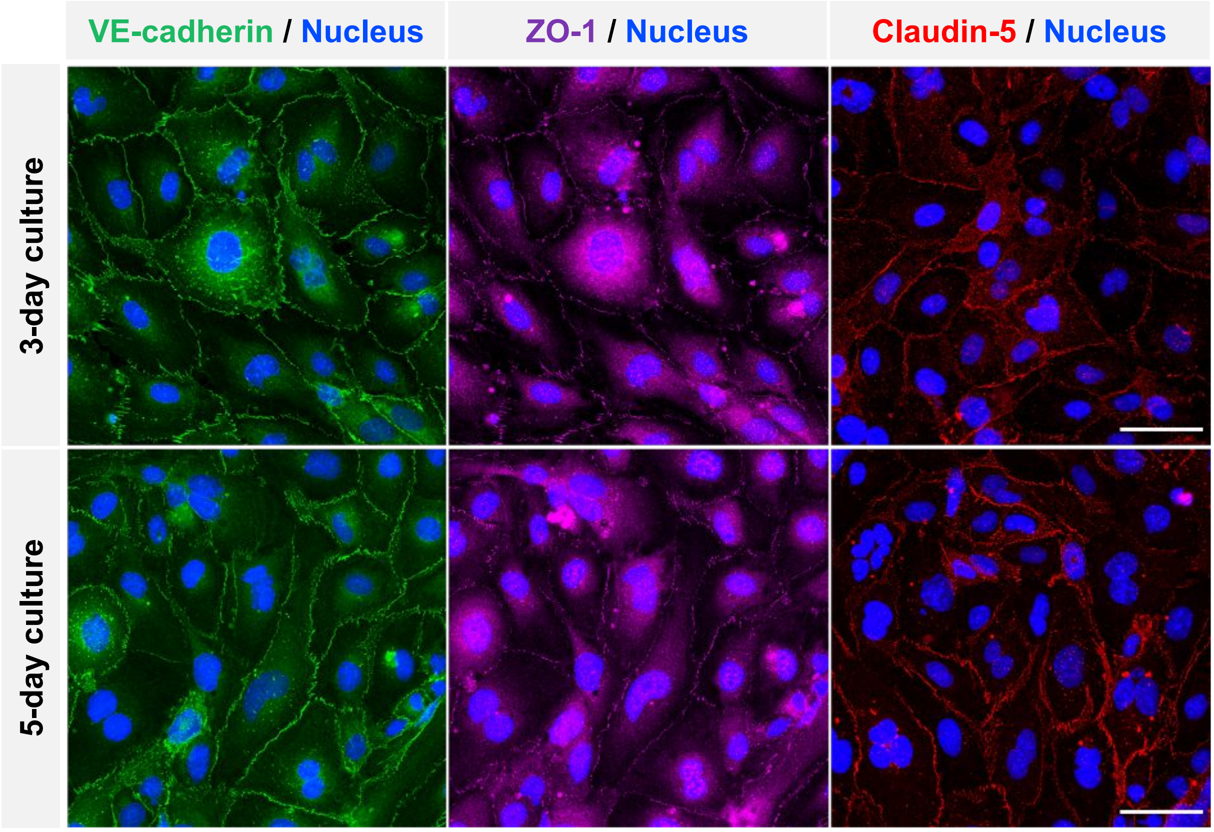

Figure. S4. Adherent and tight junction proteins localize and accumulate at cell-cell contact sites of HULEC-5a cultured as monolayers. Cells were fixed at day 3 and 5 of culture and immunostained to detect VE-cadherin (green), ZO-1 (magenta) and Claudin-5 (red). Shown are Z-maximum projections. Nuclei were stained with DAPI (blue). Scalebar is 50 μm.

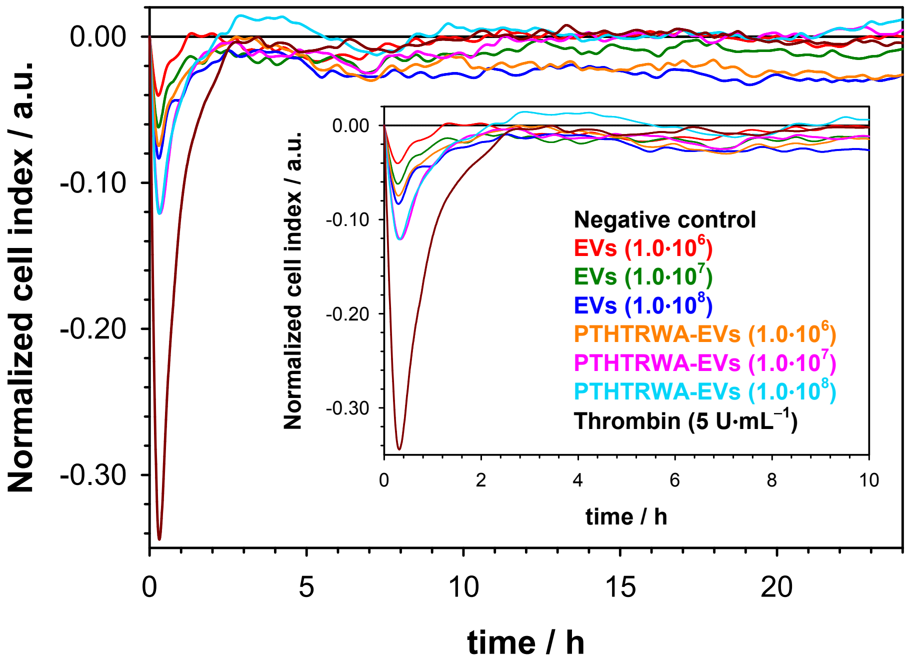

Figure. S5. Endothelial barrier integrity in HULEC-5a treated with extracellular vesicles. EVs – pristine extracellular vesicles, PTHTRWA-EVs – surface bioengineered extracellular vesicles. Cell index was normalized to baseline control. EVs and PTHTRWA-EVs are expressed in a particles·mL^{-1} units.

Alamar Blue Assay. Cytotoxicity of extracellular vesicles (EVs) decorated with and without PTHTRWA peptide was investigated in the A549 cells by the Alamar Blue assay to measure the metabolic activity of the cells after 3 and 24 hours (h) exposure to the EVs and PTHTRWA-EVs, respectively. The A549 cells (ATCC CCL-185) were seeded in 96-well plates at $1.5 \cdot 10^4$ cells·$well^{-1}$. The following day the cells were exposed to EVs (concentration: $1.7 \cdot 10^8$ particles·mL^{-1}) for 24 h. Unexposed cells were run in parallel as negative control (NC), while hydrogen peroxide, H_2O_2 (200 µM, 5 min in PBS) and methyl methanesulphonate, MMS (200 µM, 24 h exposure) was used as positive control (PC). The EVs medium was used as solvent control (SC) at the highest concentration used for exposure with EVs (concentration: $1.7 \cdot 10^8$ particles·mL^{-1}). Three independent experiments were performed, with samples run in duplicate in each experiment. At the end of exposure, the cells were washed twice with PBS and incubated for 3 h with fresh culture medium supplemented with 10% Alamar Blue staining solution. The fluorescence signal of Alamar Blue was detected on a microplate reader (FLUOstar OPTIMA, excitation 530 nm, emission 590 nm). The cell viability was calculated relative to NC cells, after subtracting the blank value (wells with only medium and Alamar Blue solution) from all wells. Possible interference of the EVs with the assay was investigated by

mixing pristine EVs (concentration:$1.7 \cdot 10^8 \cdot$ particles·mL^{-1}) with the 10% Alamar Blue staining solution in medium (without cells). No interference was detected.

Genotoxicity by Comet assay. DNA damage was investigated by the single cell gel electrophoresis/comet assay (CA). DNA strand breaks (SB) were detected by the standard alkaline comet assay, while oxidized base lesions was detected with the modified version of the assay using the enzyme formamidopyrimidine (Fpg) DNA glycosylase (kind gift from NorGenoTec AS, Norway). The A549 cells (ATCC CCL-185) were seeded in 96-well plates at a density of $1.5 \cdot 10^4$ cells·well^{-1}. The next day the cells were exposed to extracellular vesicles (EVs) decorated with and without PTHTRWA peptide (concentration: $1.7 \cdot 10^8 \cdot$particles·mL^{-1}) for 3 or 24 h. Unexposed cells were run in parallel as NC. The particles suspension medium was used as SC at the highest exposure concentration used (concentration: $1.7 \cdot 10^8 \cdot$particles·mL^{-1}). As PC for the Fpg activity, stock cells previously exposed to the photosensitizer Ro 19-8022 (2 μM, kindly provided by Hoffmann La Roche) and light irradiated were included in each experiment (A549 Ro). The selective production of DNA oxidative damage (net Fpg) was assessed in Ro 19-8022 exposed cells. As an PC for strand breaks, one slide with embedded NC cells was exposed to H_2O_2 (50 μM, Sigma-Aldrich, Germany) for 5 minutes before lysis. Three independent experiments were performed, with samples run in duplicate in each experiment. After exposure to the EVs and PTHTRWA-EVs, the cells were washed twice with PBS, detached from the plates by trypsinization, resuspended in medium and embedded in low-melting-point agarose on microscope slides. Duplica slides were prepared for all samples, one slide was used in the standard version of the CA, and one for Fpg incubation. All slides were incubated overnight in lysis solution (2.5 M NaCl; 0.1 M EDTA; 10 mM Tris; 10% v/v Triton X-100; pH 10; 4 °C) to dissolve membranes and cytoplasm and expose the nuclei. For Fpg incubation, the slides were washed 10 min × 2 in buffer F (40 mM HEPES; 0.1 M KCl; 0.5 mM EDTA; 0.2 mg·mL^{-1} BSA; pH 8; 4 °C), and incubated with the proper dilution of Fpg enzyme in a humid box at 37 °C for 30 min. All slides underwent unwinding of the supercoiled DNA by incubation in electrophoresis solution (0.3 M NaOH; 1 mM EDTA; pH > 13; 4 °C) for 20 min at 4 °C. The electrophoresis was then run in the same buffer for 20 min at 4 °C (25 V, 1.25 V·cm^{-1}, Consort EV202). Afterwards the slides were washed in PBS followed by H_2O and left to dry overnight. The nuclei were stained with SYBR gold for scoring of strand breaks (as comet tails) with a fluorescence microscope (DMI 6000 B, Leica Microsystems, Germany) equipped with a SYBR photographic filter (Thermo Fischer Scientific, USA) and the Comet Assay IV 4.3.1 software (Perceptive Instruments, UK). 50

comets per slide (100 comets per sample) were analyzed, and the median of the percentage of DNA in tail was taken as a measure of DNA damage. SBs and SBs plus oxidative DNA damage (SB + FpG) are reported. Data are presented as mean ± SD of three independent experiments.

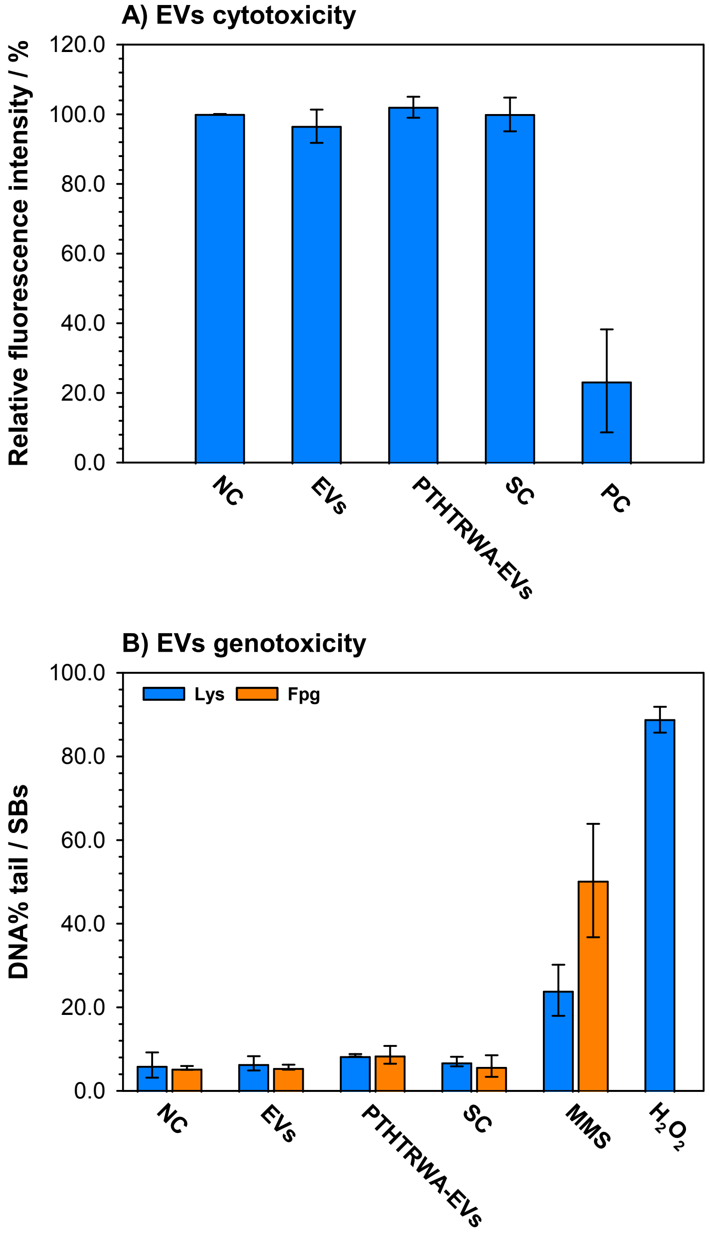

Figure. S6. (A) Alamar blue performed on A549 cells grown in 96-well plate using 10% of AB solution. Cells have been exposed to EVs for 24h after that to the AB solution for 3 h. Three independent experiments were performed with duplicate wells for each exposure. The error bars indicate the variability observed over 3 different experiments. (B) DNA strand breaks (SBs) measured by the standard alkaline comet assay (Lys) and oxidized purine bases (Fpg) measured by the enzyme (formamidopyrimidine glycosylase – Fpg) modified comet assay in A549 cells after the exposure to EVs for 24 h. The effect is expressed as tail intensity (%). Three independent experiments were performed with duplicate wells for each exposure. The results are shown as mean of the median of duplicate wells from each of the three experiments +SD, standard deviation. EVs – pristine extracellular vesicles, PTHTRWA-EVs – bioengineered extracellular vesicles, hydrogen peroxide, H_2O_2 (200 µM, 5 min in PBS) and methyl

methanesulphonate, MMS (200 µM, 24 h exposure) used as positive controls (PC), PC – positive control, NC – negative control, SC – solvent control.

6. Electroporation of superparamagnetic iron oxide nanoparticles into EVs

Iron (II,III) oxide nanoparticles, SPIO (900043 iron oxide(II,III), magnetic nanoparticles solution, Sigma -Aldrich), sized 14 nm TEM, were loaded into extracellular vesicles (EVs) using the electroporation process to fabricate the EVs-SPIO construct. Briefly, 200 µL of EVs ($4.55\cdot10^{13}$ particles$\cdot$mL^{-1}, total protein concentration measured by BCA: 4.5 mg$\cdot$mL^{-1}) were mixed with 100 µL of SPIO (5 mg$\cdot$mL^{-1}) and 4.2 mL of PBS buffer pH 7.2 with trehalose (50 mM). Square wave electroporation was carried out in 4 mm cuvettes using a BTX ECM830 electroporator (Harvard Bioscience Inc, USA) setup for 400 V with 1 ms pulse. To restore the EVs membrane, the post-electroporation suspension was incubated at room temperature for 24 h in a dark place. The SPIO-loaded EVs were washed twice in PBS following ultracentrifugation (100,000 g) in a Beckman JA-30.50 Ti rotor for 1.5 h. The supernatant was removed, and the pellets were resuspended in PBS at pH 7.2 to a final protein concentration of 0.2 mg$\cdot$mL^{-1}.[11] The SPIO-loaded EVs prepared in this way were subjected to surface functionalization with targeting heptapeptide PTHTRWA (please see chapter 2.3 in the main text).

REFERENCES

(1) Marshall, G. R.; Merrifield, R. B. Synthesis of Angiotensins by the Solid-Phase Method. *Biochemistry* **1965**, *4* (11), 2394–2401. https://doi.org/10.1021/bi00887a020.
(2) Merrifield, R. B. Solid-Phase Peptide Synthesis. III. An Improved Synthesis of Bradykinin. *Biochemistry* **1964**, *3* (9), 1385–1390. https://doi.org/10.1021/bi00897a032.
(3) Merrifield, R. B. Solid Phase Peptide Synthesis. I. The Synthesis of a Tetrapeptide. *J Am Chem Soc* **1963**, *85* (14), 2149–2154. https://doi.org/10.1021/ja00897a025.
(4) Gisin, B. F. The Preparation of Merrifield-Resins Through Total Esterification With Cesium Salts. *Helv Chim Acta* **1973**, *56* (5), 1476–1482. https://doi.org/10.1002/hlca.19730560503.
(5) Ruzycka-Ayoush, M.; Nowicka, A. M.; Kowalczyk, A.; Gluchowska, A.; Targonska, A.; Mosieniak, G.; Sobczak, K.; Donten, M.; Grudzinski, I. P. Exosomes Derived from Lung Cancer Cells: Isolation, Characterization, and Stability Studies. *European Journal of Pharmaceutical Sciences* **2023**, *181*, 106369. https://doi.org/10.1016/j.ejps.2022.106369.
(6) Discovery Studio v.21 Interfaces BIOVIA. Bild and Edit Protein. 2021.
(7) Frisch, M. J.; Trucks, G. W.; Schlegel, H. B.; Scuseria, G. E.; Robb, M. A.; Cheeseman, J. R.; Scalmani, G.; Barone, V.; Petersson, G. A.; Nakatsuji, H.; Li, X.; Caricato, M.; Marenich, A. V.; Bloino, J.; Janesko, B. G.; Gomperts, R.; Mennucci, B.; Hratchian, H. P.; Ortiz, J. V.; Izmaylov, A. F.; Sonnenberg, J. L.; Williams-Young, D.; Ding, F.; Lipparini, F.; Egidi, F.; Goings, J.; Peng, B.; Petrone, A.; Henderson, T.;

Ranasinghe, D.; Zakrzewski, V. G.; Gao, J.; Rega, N.; Zheng, G.; Liang, W.; Hada, M.; Ehara, M.; Toyota, K.; Fukuda, R.; Hasegawa, J.; Ishida, M.; Nakajima, T.; Honda, Y.; Kitao, O.; Nakai, H.; Vreven, T.; Throssell, K.; Montgomery, J. A. , Jr.; Peralta, J. E.; Ogliaro, F.; Bearpark, M. J.; Heyd, J. J.; Brothers, E. N.; Kudin, K. N.; Staroverov, V. N.; Keith, T. A.; Kobayashi, R.; Normand, J.; Raghavachari, K.; Rendell, A. P.; Burant, J. C.; Iyengar, S. S.; Tomasi, J.; Cossi, M.; Millam, J. M.; Klene, M.; Adamo, C.; Cammi, R.; Ochterski, J. W.; Martin, R. L.; Morokuma, K.; Farkas, O.; Foresman, J. B.; Fox, D. J. Gaussian 16, Gaussian, Inc., Wallingford CT,. 2016.

(8) Breneman, C. M.; Wiberg, K. B. Determining Atom-centered Monopoles from Molecular Electrostatic Potentials. The Need for High Sampling Density in Formamide Conformational Analysis. *J Comput Chem* **1990**, *11* (3), 361–373. https://doi.org/10.1002/jcc.540110311.

(9) Nagae, M.; Re, S.; Mihara, E.; Nogi, T.; Sugita, Y.; Takagi, J. Crystal Structure of A5β1 Integrin Ectodomain: Atomic Details of the Fibronectin Receptor. *Journal of Cell Biology* **2012**, *197* (1), 131–140. https://doi.org/10.1083/jcb.201111077.

(10) Wu, G.; Robertson, D. H.; Brooks, C. L.; Vieth, M. Detailed Analysis of Grid-based Molecular Docking: A Case Study of CDOCKER—A CHARMm-based MD Docking Algorithm. *J Comput Chem* **2003**, *24* (13), 1549–1562. https://doi.org/10.1002/jcc.10306.

(11) Jia, G.; Han, Y.; An, Y.; Ding, Y.; He, C.; Wang, X.; Tang, Q. NRP-1 Targeted and Cargo-Loaded Exosomes Facilitate Simultaneous Imaging and Therapy of Glioma in Vitro and in Vivo. *Biomaterials* **2018**, *178*, 302–316. https://doi.org/10.1016/j.biomaterials.2018.06.029.
